# Transcriptomic signatures induced by the Ebola virus vaccine rVSVΔG-ZEBOV-GP in adult cohorts in Europe, Africa, and North America: a molecular biomarker study

**DOI:** 10.1016/S2666-5247(21)00235-4

**Published:** 2021-12-06

**Authors:** Eleonora Vianello, Patricia Gonzalez-Dias, Suzanne van Veen, Carmen G Engele, Edwin Quinten, Thomas P Monath, Donata Medaglini, VSV-EBOVAC Consortium, Selidji T Agnandji, Rafi Ahmed, Jenna Anderson, Floriane Auderset, Philip Bejon, Luisa Borgianni, Jessica Brosnahan, Annalisa Ciabattini, Olivier Engler, Mariëlle C Haks, Ali M Harandi, Donald Gray Heppner, Alice Gerlini, Angela Huttner, Peter G Kremsner, Donata Medaglini, Thomas P Monath, Francis M Ndungu, Patricia Njuguna, Tom H M Ottenhoff, David Pejoski, Mark Page, Gianni Pozzi, Francesco Santoro, Claire-Anne Siegrist, VSV-EBOPLUS Consortium, Selidji T Agnandji, Luisa Borgianni, Annalisa Ciabattini, Sheri Dubey, Michael Eichberg, Olivier Engler, Alice Gerlini, Patricia Gonzales Dias Carvalho, Mariëlle C Haks, Ali M Harandi, Angela Huttner, Peter G Kremsner, Kabwende Lumeka, Donata Medaglini, Helder I Nakaya, Sravya S Nakka, Essone P Ndong, Tom H M Ottenhoff, Gianni Pozzi, Sylvia Rothenberger, Francesco Santoro, Claire-Anne Siegrist, Suzanne van Veen, Eleonora Vianello, Francesco Santoro, Angela Huttner, Sheri Dubey, Michael Eichberg, Francis M Ndungu, Peter G Kremsner, Paulin N Essone, Selidji Todagbe Agnandji, Claire-Anne Siegrist, Helder I Nakaya, Tom H M Ottenhoff, Mariëlle C Haks

**Affiliations:** Department of Infectious Diseases, Leiden University Medical Center, Leiden, Netherlands; Department of Clinical and Toxicological Analyses, School of Pharmaceutical Sciences, University of São Paulo, São Paulo, Brazil; NewLink Genetics Corporation, Devens, MA, USA; Laboratory of Molecular Microbiology and Biotechnology, Department of Medical Biotechnologies, University of Siena, Siena, Italy; Sclavo Vaccines Association, Siena, Italy; Division of Infectious Diseases; Center for Vaccinology; Geneva University Hospitals and Faculty of Medicine, Geneva, Switzerland; Department of Vaccine and Biologics Research, Merck Research Laboratories, West Point, PA, USA; Department of Biosciences, KEMRI-Wellcome Trust Research Programme, Kilifi, Kenya; Centre de Recherches Médicales de Lambaréné, Lambaréné, Gabon; Institut für Tropenmedizin, Universitätsklinikum Tübingen, and German Center for Infection Research, Tübingen, Germany; Scientific Platform Pasteur-USP, São Paulo, Brazil

## Abstract

**Background:**

A recombinant vesicular stomatitis virus vector expressing the Zaire Ebola virus glycoprotein (rVSVΔG-ZEBOV-GP) vaccine has been reported as safe, immunogenic, and highly protective in a ring vaccination trial. We aimed to identify transcriptomic immune response biomarker signatures induced by vaccination and associated signatures with its immunogenicity and reactogenicity to better understand the potential mechanisms of action of the vaccine.

**Methods:**

354 healthy adult volunteers were vaccinated in randomised, double-blind, placebo-controlled trials in Europe (Geneva, Switzerland [November, 2014, to January, 2015]) and North America (USA [Dec 5, 2014, to June 23, 2015]), and dose-escalation trials in Africa (Lambaréné, Gabon [November, 2014, to January, 2015], and Kilifi, Kenya [December, 2014, to January, 2015]) using different doses of the recombinant vesicular stomatitis virus vector expressing the Zaire Ebola virus glycoprotein (rVSVΔG-ZEBOV-GP; 3 × 10^5^ to 1 × 10^8^ plaque-forming units [pfu]). Longitudinal transcriptomic responses (days 0, 1, 2, 3, 7, 14, and 28) were measured in whole blood using a targeted gene expression profiling platform (dual-colour reverse-transcriptase multiplex ligation-dependent probe amplification) focusing on 144 immune-related genes. The effect of time and dose on transcriptomic response was also assessed. Logistic regression with lasso regularisation was applied to identify host signatures with optimal discriminatory capability of vaccination at day 1 or day 7 versus baseline, whereas random-effects models and recursive feature elimination combined with regularised logistic regression were used to associate signatures with immunogenicity and reactogenicity.

**Findings:**

Our results indicated that perturbation of gene expression peaked on day 1 and returned to baseline levels between day 7 and day 28. The magnitude of the response was dose-dependent, with vaccinees receiving a high dose (≥9 × 10^6^ pfu) of rVSVΔG-ZEBOV-GP exhibiting the largest amplitude. The most differentially expressed genes that were significantly upregulated following vaccination consisted of type I and II interferon-related genes and myeloid cell-associated markers, whereas T cell, natural killer cell, and cytotoxicity-associated genes were downregulated. A gene signature associated with immunogenicity (common to all four cohorts) was identified correlating gene expression profiles with ZEBOV-GP antibody titres and a gene signatures associated with reactogenicity (Geneva cohort) was identified correlating gene expression profiles with an adverse event (ie, arthritis).

**Interpretation:**

Collectively, our results identify and cross-validate immune-related transcriptomic signatures induced by rVSVΔG-ZEBOV-GP vaccination in four cohorts of adult participants from different genetic and geographical backgrounds. These signatures will aid in the rational development, testing, and evaluation of novel vaccines and will allow evaluation of the effect of host factors such as age, co-infection, and comorbidity on responses to vaccines.

**Funding:**

Innovative Medicines Initiative 2 Joint Undertaking.

## Introduction

Ebola virus disease is a rare but severe infectious disease which is caused by Ebola virus. Ebola virus disease is characterised by a high fatality rate (average of 50%, up to 90%) and can result in uncontrolled epidemics. The severe 2014–16 Ebola virus disease outbreak in west Africa registered more than 11 300 deaths among 30 000 suspected, probable, and confirmed cases. Moreover, the recent epidemic in the Democratic Republic of Congo, with an approximate 65% fatality rate among more than 3000 confirmed Ebola virus disease cases, confirms the urgency for protective vaccines to prevent disease spread.^1^

The recombinant vesicular stomatitis virus vector expressing the Zaire Ebola virus glycoprotein (rVSVΔGZEBOV-GP) is a live-attenuated vaccine in which the vesicular stomatitis virus glycoprotein encoding gene has been replaced with the Zaire Ebola virus glycoprotein (ZEBOV-GP).^2^ Challenge studies in animal models showed high efficacy and specificity of the rVSVΔG-ZEBOV-GP vaccine against Ebola virus,^3–7^ and numerous clinical trials (phase 1–3) in North America, Europe, and Africa,^7,8^ provided further evidence that the vaccine is safe, efficacious, and fast-acting after a single injection. Moreover, a phase 3 ring vaccination trial of rVSVΔG-ZEBOV-GP was successfully completed in Guinea during an outbreak in 2015, confirming vaccine efficacy after a single injection.^8^ More recently, in 2018–19 in the Democratic Republic of the Congo, over 250 000 people were vaccinated in a ring vaccination approach using rVSVΔG-ZEBOV-GP in an expanded access (also known as compassionate use) protocol. Preliminary data corroborated earlier findings from the ring vaccination trial in Guinea that rVSVΔG-ZEBOV-GP confers rapid and robust protection, reinforcing its suitability in outbreak situations.^9^ As a result, the procedure for licensing the vaccine was markedly accelerated leading to its licensure by the European Medicines Agency (Jan 14, 2021) and the US Food and Drug Administration (Dec 19, 2019) as well as prequalification by WHO (Nov 12, 2019).^10–12^

Although the rVSVΔG-ZEBOV-GP vaccine is highly effective against Ebola virus, only a few studies have explored its underlying immune mechanisms and its ability to induce long-term protection.^13^ An innate (non-specific) response-associated signature was identified in a European cohort (Geneva, Switzerland) and validated in an African cohort (Lambaréné, Gabon).^14^ This signature was vaccine dose-dependent and correlated with viraemia and adverse events, including arthritis. Additionally, the analysis of rVSVΔG-ZEBOV-GP-specific adaptive cellular and humoral responses revealed strong B-cell responses together with potent Ebola virus-neutralising antibodies^15^ whereas the overall T-cell responses were weak to moderate.^16^ To further unravel the mode of action of the rVSVΔG-ZEBOV-GP vaccine, we investigated at the transcriptional level the longitudinal and dose-dependent kinetics of the innate and adaptive immune responses following vaccination of healthy volunteers in Europe, North America, and Africa.

## Methods

### Study design and participants

We did a pre-planned transcriptomic analysis on blood samples collected during four clinical trials testing the safety and immunogenicity of the rVSVΔG-ZEBOV-GP vaccine. The trials were conducted in Europe (phase 1/2, randomised, double-blind, placebo-controlled, dose-finding trial in Geneva, Switzerland [November, 2014, to January, 2015; NCT02287480]), North America (phase 1b, randomised, double-blind, placebo-controlled, dose-response trial in the USA [Dec 5, 2014, to June 23, 2015; NCT02314923]), Africa (phase 1, randomised, open-label, dose-escalation trial in Lambaréné, Gabon [November, 2014, to January, 2015; PACTR201411000919191] and phase 1, open-label, dose-escalation trial in Kilifi, Kenya [December, 2014, to January, 2015; NCT02296983]). Details of the study design and participant groups have been summarised in the appendix 1. All participants provided written informed consent. The trial protocols were reviewed and approved by the WHO’s Ethics Committee as well as by local ethics committees (appendix 2 p 4).

### Procedures and outcomes

**Figure 1:**
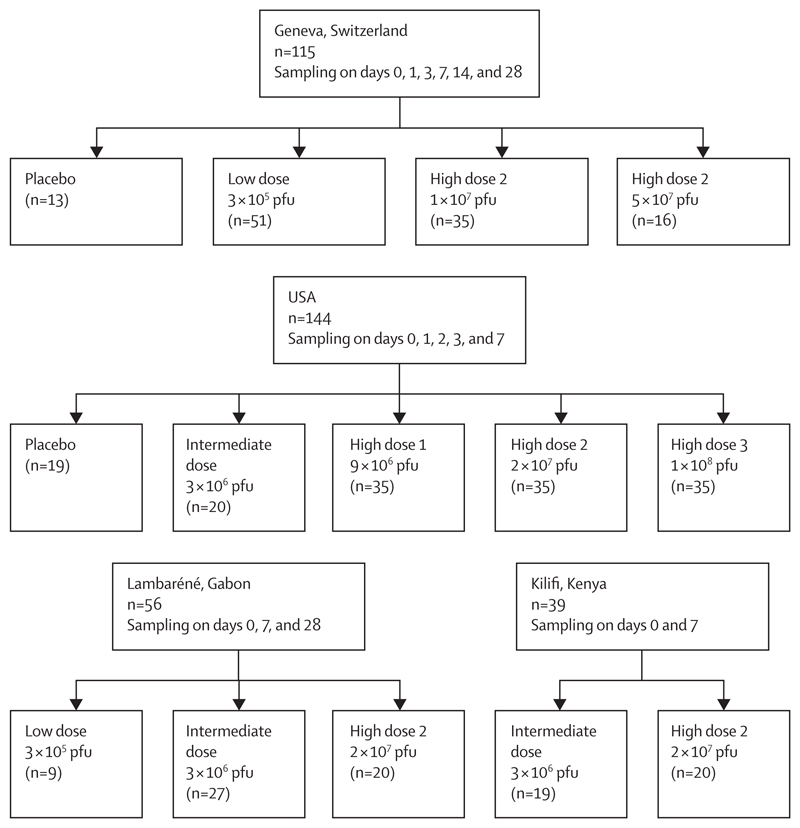
Flow diagram of the four study cohorts Overview of the number of participants in each study, of the timepoints at which peripheral whole blood samples were collected with day 0 as baseline before vaccination, and the number of participants who were given the various vaccine doses or placebo. pfu=plaque-forming units.

2·5 mL venous blood was collected in PAXgene blood RNA tubes (PreAnalytiX, Hombrechtikon, Switzerland) from all participants (on day 0, 1, 2, 3, 7, 14, and 28; dose of 3 × 10^5^ − 1 × 10⁸ plaque-forming units [pfu]; figure 1). RNA was isolated using the PAXgene blood miRNA kit (PreAnalytiX) according to the manufacturer’s automated protocol including on-column DNase digestion. RNA yield was quantified with a Qubit fluorometer (ThermoFisher Scientific, Wilmington, DE, USA) using an RNA Broad Range assay Kit (ThermoFisher Scientific). Gene expression profiling was performed using dual-colour reverse-transcriptase multiplex ligation-dependent probe amplification (dcRT-MLPA; appendix 2 p 4),^17^ comprising four housekeeping genes and 144 selected immune-related genes (appendix 3). ZEBOV-GP-specific IgG enzyme-linked immunosorbent assay antibody titres have been previously collected (appendix 2 p 5).^18–20^

### Statistical analysis

*GAPDH*-normalised log_2_-transformed gene expression levels were used for differential expression analysis for each cohort. For statistical significance, the non-parametric Mann-Whitney *U* test with Benjamini-Hochberg correction for multiple testing was applied. A p value of less than 0·05 and log_2_-fold changes of less than –0·6 and more than 0·6 were set as thresholds for the identification of differentially expressed genes (DEGs).

Ingenuity pathway analysis (IPA-60467501; QIAGEN, Hilden, Germany) was used to explore interactive networks between the DEGs of each cohort. To evaluate the effect of time and dose on the gene expression perturbation, molecular degree of perturbation (R package mdp) and principal component analysis (function prcomp from R package stats) were used. Signatures with the best discriminatory capability of vaccination at day 1 or day 7 versus baseline were identified using logistic regression with lasso regularisation. Leave-one-out cross-validation and train-test split were used to assess the performance of the trained regression models. When pooling cohorts, a random down-sampling approach was applied (if required) to obtain a balanced distribution of the number of individuals within the pooled cohort. To identify immunogenicity-associated signatures, gene expression data were correlated with ZEBOV-GP-specific IgG antibody titres using Spearman correlation in each cohort. Significant correlations obtained in single cohorts (p<0·05) were subsequently integrated using the random-effects models for meta-analyses (R package metaphor). Arthritis was selected for correlation with reactogenicity because of its late onset (day 10–14 post-injection) and prolonged duration (>1 week), compared with other adverse events (ie, pain at the injection site, fever, nausea, and fatigue). Moreover, our analysis was limited to the Geneva cohort, because the low frequency of individuals developing arthritis in the other cohorts precluded comparable analyses (appendix 2 p 6). Samples were split into training (70%) and test (30%) sets. Recursive feature elimination (R package caret) was applied to the training set to select the top-ranking genes able to distinguish vaccinees who developed arthritis from those who did not. Subsequently, different machine learning algorithms available on the caret R package were trained and evaluated by cross-validation (5 k-fold). The classifying performance of the model was assessed by evaluating sensitivity, specificity, receiver operating characteristic (ROC) curve, and area under the ROC curve (AUC) with 95% CI. Details of these analyses are available in appendix 2 (pp 4–6). Data were analysed in R (version 3.5.1).

### Role of the funding source

The funder had no role in study design, data collection, data analysis, data interpretation, or writing of the report.

## Results

**Figure 2:**
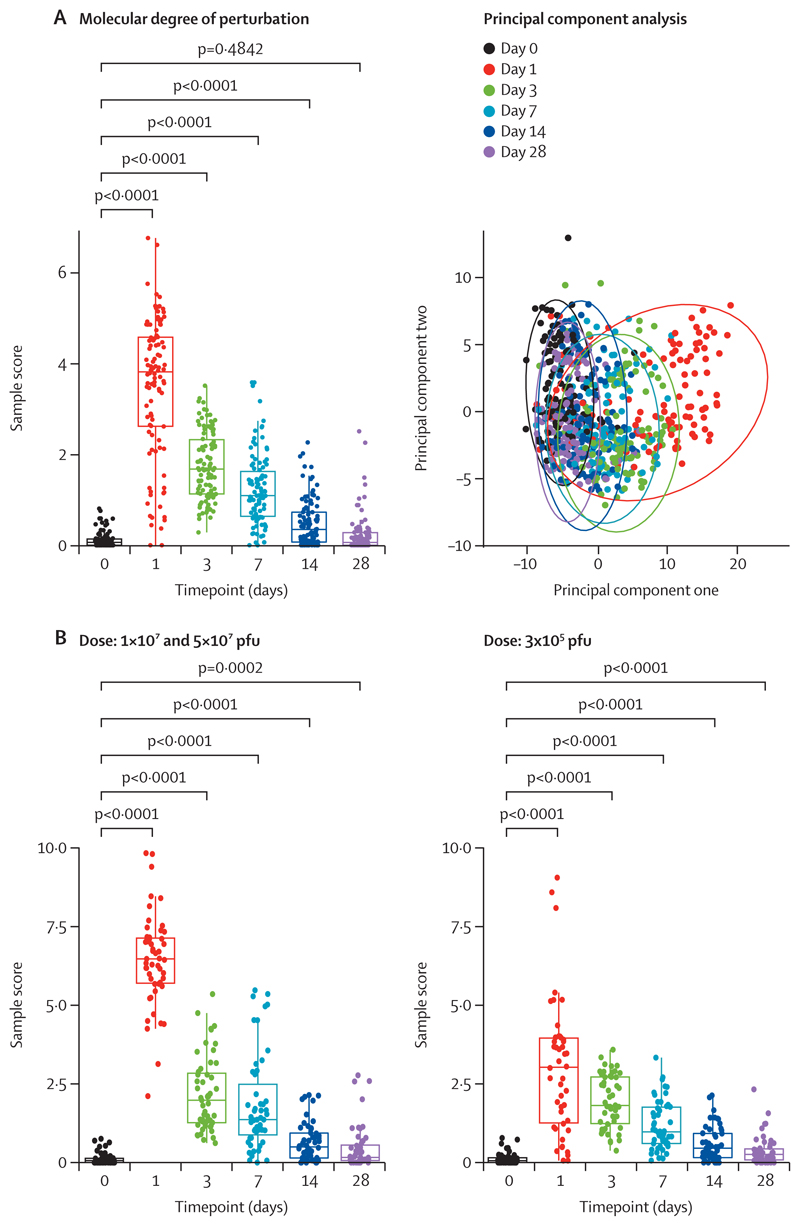
Effect of time and dose on gene expression profiles after rVSVΔG-ZEBOV-GP vaccination in the Geneva cohort (A) Molecular degree of perturbation and principal component analysis performed on *GAPDH*-normalised, log_2_-transformed gene expression data of the Geneva cohort to evaluate the effect of time by separating samples by timepoint (days 0, 1, 3, 7, 14, and 28). Day 0 samples of vaccinees were used as baseline controls. Timepoints were compared using Mann-Whitney *U* test. (B) Effect of dose (1 × 10^7^ and 5 × 10^7^ pfu *vs* 3 × 10^5^ pfu) evaluated by molecular degree of perturbation analysis at distinct timepoints. Day 0 samples of vaccinees were used as baseline controls. Timepoints were compared using Mann-Whitney *U* test. pfu=plaque-forming units. rVSVΔG-ZEBOV-GP=recombinant vesicular stomatitis virus vector expressing the Zaire Ebola virus glycoprotein.

We investigated the longitudinal transcriptomic profiles in response to rVSVΔG-ZEBOV-GP vaccination in four cohorts from different genetic and geographical backgrounds (Switzerland, USA, Gabon, and Kenya; figure 1; appendix 1).^18–20^ We first evaluated host gene expression kinetics in the Geneva (Switzerland) cohort (appendix 4).^18^ Principal component analysis and molecular degree of perturbation analyses showed that gene expression perturbation peaked at day 1 after vaccination and gradually returned to basal levels at day 28 (figure 2A). Although the kinetics of the overall transcriptomic response was dose-independent, the magnitude of the vaccine response was dose-dependent, with larger gene expression perturbations in participants who received a high dose (high dose 2 is 1 × 10⁷ pfu and 5 × 10⁷ pfu; figure 2B). Moreover, high-dose-2 vaccinees distinctly clustered at day 1 when compared with their own baseline or to placebo controls (appendix 2 p 7), whereas low-dose vaccinees (3 × 10^5^ pfu) did not exhibit a complete separation until day 3 compared with their own baseline, suggesting slower kinetics in low-dose vaccinees.

**Figure 3:**
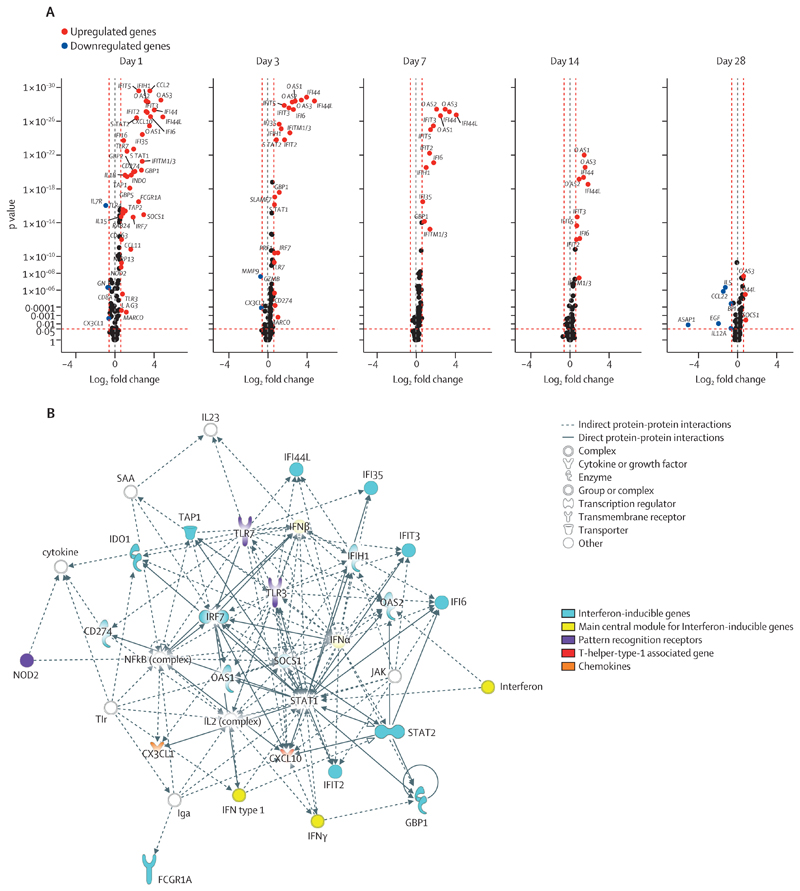
Identification of DEGs and key networks after rVSVΔG-ZEBOV-GP vaccination in the Geneva cohort Differential expression analysis was performed on *GAPDH*-normalised log_2_-transformed gene expression data of the Geneva cohort. (A) Volcano plots representing DEGs at different timepoints (days 1, 3, 7, 14, and 28) after rVSVΔG-ZEBOV-GP vaccination of all vaccinees (high dose 2 plus low dose) compared with their baseline gene expression levels. The y-axis scales of all plots are harmonised. p values are shown on a –log_10_ scale for better visualisation. Genes with p<0·05 and log_2_ fold change of less than –0·6 or more than 0·6 were labelled as DEGs. (B) Ingenuity pathway analysis interactive network analysis of DEGs identified between day 0 and day 1 following rVSVΔG-ZEBOV-GP vaccination of all vaccinees (high dose two and low dose) compared with their baseline gene expression levels. The shapes of the nodes represent the functional classes of the gene products. DEG=differentially expressed gene. *IFITM_1/3_*=*IFITM_1_* or *IFITM_3_*, or both. rVSVΔG-ZEBOV-GP=recombinant vesicular stomatitis virus vector expressing the Zaire Ebola virus glycoprotein.

Next, we identified DEGs in response to vaccination. When comparing vaccinees at different timepoints after vaccination versus their own baseline, we verified that most transcripts were differentially expressed at day 1 (39 upregulated, p<0·0001 in 37 [95%] of 39 DEGs, and four downregulated, p<0·0001 in three [75%] of four DEGs); gene expression perturbation was transient and gradually returned to baseline levels at day 28; and the magnitude of the vaccine-response was dose-dependent (figure 3A; appendix 2 p 8; appendix 5). Similar results were observed comparing vaccinees (high dose 2 plus low dose) versus participants who received placebo (appendix 2 p 9; appendix 5), further corroborating the prompt and dose-dependent peak of the transcriptomic response. Interactive network analysis indicated that most of the upregulated DEGs at day 1 post-vaccination belong to the interferon (IFN)-signalling genes (ISGs; figure 3). Additionally, a T-helper-type-1 (Th1) associated gene (ie, *CXCL10*), pattern-recognition receptors (ie, *TLR3*, *TLR7*, and *NOD2*), and the myeloid subset marker *CD163* were also significantly upregulated. By contrast, the downregulated DEGs included primarily T-cell markers (ie, *CD8A* and *IL7R*) and the cytotoxicity marker *GNLY*, whereas other T-cells markers (ie, *CD4* and *CD3E*), cytotoxicity markers (ie, *GZMA*), and natural killer (NK)-cell markers (ie, *NCAM*) were downregulated without reaching statistical significance (appendix 2 p 10; appendix 5). The DEGs network identified between high-dose-2 and low-dose vaccinees at day 1 after vaccination was similarly dominated by ISGs, but also included several myeloid-associated genes (ie, *FCGR1A*, *MARCO*, *IL12A*, *IL12B*, and *CCL3*) of which *CCL3* remained differentially expressed until day 28 (appendix 2 pp 8, 12). Detailed analysis of the individual DEGs kinetic profiles revealed two subgroups: fast-kinetics genes, which normalised to baseline levels by day 3 after the peak response at day 1, and slow-kinetics genes, which did not return to baseline levels until day 14–28. Although most DEGs showed fast kinetics (ie, *CXCL10*, *FCGR1A*, *GBP2*, and *GBP5*), several ISGs displayed slow kinetics (ie, *OAS3*, *IFI6*, *OAS2*, *IFI44*, and *IFI44L*). Interestingly, together with a lower response magnitude, several genes in low-dose vaccinees displayed slower expression kinetics than in the high-dose-2 vaccinees, with ISGs preferably displaying a peak response at day 3 instead of day 1 (ie, *IFI44L*, *IFI44, IFI6, OAS2*, and *OAS3*; appendix 2 p 10).

We then aimed to validate the findings of the Geneva cohort in independent cohorts with distinct geographical and genetic backgrounds (appendix 4).^20^ Similarly, in the USA cohort, both molecular degree of perturbation and principal component analysis analyses identified the transcriptomic perturbation peak at day 1 after vaccination and a dose-dependent response magnitude, evidenced by less gene perturbation with intermediate-dose (3 × 10^6^ pfu) than with high dose 1 (9 × 10^6^ pfu), high dose 2 (2 × 10^7^ pfu; currently used for clinical application), and high dose 3 (1 × 10^8^ pfu; appendix 2 p 13). No discrimination between the high-dose groups were observed, suggesting similar transcriptomic profiles in doses of 9 × 10^6^ pfu or higher. Differential expression analysis by comparing all vaccinees at different timepoints post-vaccination versus their own baseline confirmed that most of DEGs were identified at day 1 post-vaccination (28 upregulated, p<0·0001; five downregulated, with p<0·0001 for four [80%] of the five DEGs); most DEGs were transiently regulated and gradually return to baseline levels over the next 7 days; and the magnitude of the response was dose-dependent (appendix 2 p 14; appendix 5). Like in the Geneva cohort, ISGs were overrepresented among the day-1-upregulated DEGs (appendix 2 p 14), including both slow-kinetics genes (ie, *IFI6*, *OAS2*, *OAS3*, *IFI44*, and *IFI44L*) and fast-kinetics genes (ie, *CXCL10*, *FCGR1A*, and *GBP2*). Differently to the Geneva cohort, *GBP5* showed a slow kinetics (appendix 2 p 16). Although we could not detect any perturbation of NK-cell marker *NCAM1* at day 1, several T-cell and cytotoxicity markers were significantly downregulated, whereas the myeloid-associated marker *CD14* was significantly upregulated, further corroborating the findings in the Geneva cohort (appendix 2 p 16). In general, a high proportion of DEGs (25 [49%] of 51 at day 1) were shared between Geneva and USA cohorts (appendix 2 p 18).

**Figure 4:**
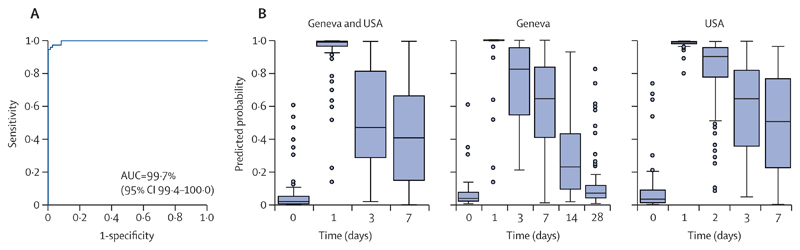
Identification of signatures associated with rVSVΔG-ZEBOV-GP vaccination at the peak of the transcriptomic response (day 1) in a pooled dataset of the Geneva and USA cohorts The pooled dataset of the Geneva and USA cohorts was used to train the model in which 70% of each dataset was used as training set and the remaining 30% of each dataset was used as test set. (A) Receiver operating characteristic curve and AUC show the classifying performance of the trained model. (B) Predicted probability plots showing the accuracy of the identified pooled biomarker signature across timepoints in box-and-whiskers plots (5–95 percentiles) either in the pooled cohort in which train-test split was performed or in the single validation cohorts. AUC=area under the receiver operating characteristic curve. rVSVΔG-ZEBOV-GP=recombinant vesicular stomatitis virus vector expressing the Zaire Ebola virus glycoprotein.

We next validated our findings in two smaller African cohorts (Lambaréné [Gabon] and Kilifi [Kenya]; appendix 4).^19^ Because in both African cohorts the earliest samples for RNA expression analysis were collected at day 7 after vaccination, we could not capture the early peak of the transcriptomic response. Nevertheless, consistent with our previous findings, principal component analysis and molecular degree of perturbation analyses in Lambaréné and Kilifi revealed a partial separation between day-7 transcriptomic profiles with their baseline values, with a concomitant dose-dependent degree of gene perturbation, whereas day 28-transcriptomic profiles were almost close to baseline levels (appendix 2 pp 19, 23). Differential expression analysis at day 7 post-vaccination identified 37 DEGs (34 upregulated, p<0·0001 in 16 [47%] of 34 DEGs; three downregulated, p<0·010 in two [67%] of three DEGs) in the Lambaréné cohort and 13 DEGs (12 upregulated, p<0·0001 in 10 [83%] of 12 DEGs; one downregulated, p<0·0033) in the Kilifi cohort. Consistent with our findings in the Geneva and USA cohorts, ISGs were overrepresented, in particular those exhibiting a slow kinetic response (appendix 2 pp 19–25; appendix 5). The eight genes shared between the four cohorts are classified as slow-kinetic-ISGs (*IFI44L*, *IFI44*, *IFI6*, *IFITM1* or *IFITM3* [or both], *OAS2*, *OAS3*, *IFIT2*, and *IFIT3*; appendix 2 p 18). Additionally, several pattern-recognition receptors (ie, *TLR1*, *TLR3*, and *CLEC7A*), Th1-associated genes (ie, *IL15* and *TBX21*), and Th2-associated genes (ie, *GATA3* and *IL5*) were detected in the Lambaréné cohort (appendix 2 p 19). No DEGs were detected in the Lambaréné cohort at day 28 post-vaccination. To identify common host biomarker signatures associated with rVSVΔG-ZEBOV-GP vaccination irrespective of population heterogeneity, we applied logistic regression with lasso regularisation at the peak of the transcriptomic response (day 1) in the Geneva and USA cohorts. The biomarker signatures that classified participants before versus after vaccination with the highest discriminatory power were composed of two genes (*CCL2* and *IFIT5*) for the Geneva cohort and five genes (*CXCL10*, *IFI44L*, *IFI6*, *IFIT2*, and *OAS3*)for the USA cohort (appendix 6). ROC curves displayed the classifying capability of these biomarker signatures in either the test set (30% of the remaining dataset of the same cohort) or the validation set (the complete dataset of the other cohort; appendix 2 p 27). The identified biomarker signatures have excellent discriminatory values both in the test and the validation sets (AUC=91·0–99·8%). Next, we evaluated the ability of the signatures identified at day 1 to classify vaccinees at later timepoints. The signatures’ discriminatory power declined over time when using the identified host biomarker signatures as classifiers (appendix 2 p 27; appendix 7), coinciding with normalisation of gene expression levels during this time window (figure 2A; appendix 2 p 13). Pooling the datasets of the two cohorts resulted in a combination of the two cohort-specific signatures that slightly affected the performance of the model (AUC 99·7%, 95% CI 99·4–100·0) and the predicted probability (figure 4; appendix 6; appendix 7).

We next set out to identify common vaccination-associated signatures in all four cohorts at day 7 post-vaccination (the earliest timepoint shared by all cohorts). Biomarker signatures at day 7 post-vaccination with the highest discriminatory power encompassed eight genes in the Geneva cohort (*CCL3*, *CD163*, *GZMA*, *IFI44*, *OAS1*, *OAS2*, *RAB33A*, and *STAT1*) and seven genes in the USA cohort (*HCK*, *IFI44*, *IFI44L*, *IFITM3*, *OAS2*, *OAS3*, and *PRF1*; appendix 6). The classification performance of both signatures was excellent in all four cohorts (AUC=85·9–99·7%; appendix 2 p 28). Exploring the capability of the signatures identified at day 7 to classify vaccinees over a broader range of timepoints after vaccination, a marked improvement in the discriminatory power was observed when signatures were evaluated in the test sets. However, the classification performance of the day-7 signatures in the validation sets was cohort-dependent. Although the Geneva day-7 signature displayed high discriminatory power in the Kilifi cohort, the USA day-7 signature outperformed the Geneva signature in classifying vaccinees before versus after vaccination in the Lambaréné cohort (appendix 2 p 28). These results were also reflected in the F1-scores (harmonic mean of precision and recall; appendix 8), indicating a bias in the capability of the model to discriminate among the classes.

**Figure 5:**
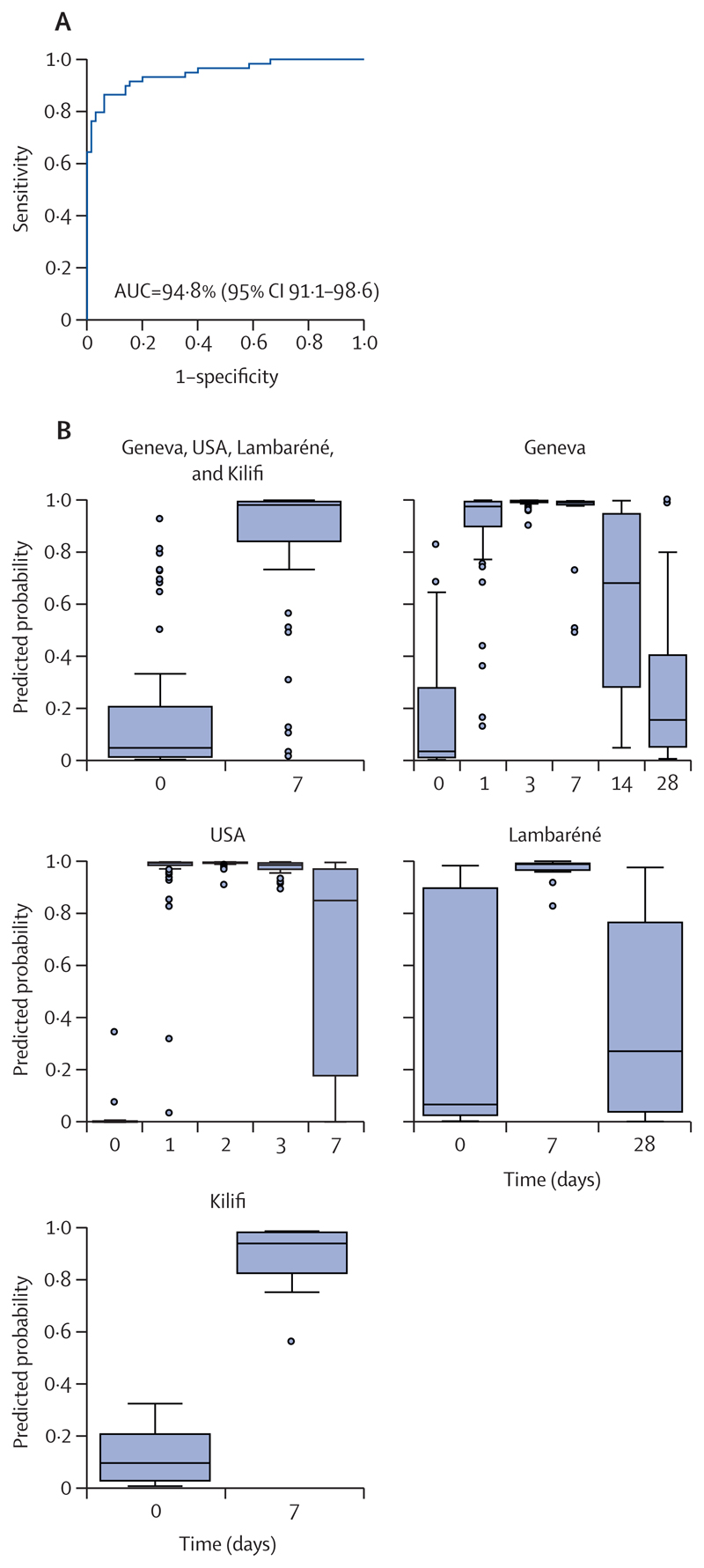
Identification of signatures associated with rVSVΔG-ZEBOV-GP vaccination at day 7 in a balanced pooled dataset of all four cohorts The balanced pooled dataset of the four cohorts was used to train the model in which 70% of each dataset was used as train set and the remaining 30% of each set was used as test set. (A) Receiver operating characteristic curve and AUC show the classifying performance of the trained model. (B) Predicted probability plots showing the accuracy of the identified pooled biomarker signature across timepoints in box-and-whiskers plots (5–95 percentiles) either in the pooled cohort in which train-test split was performed or in the single validation cohorts. AUC=area under the receiver operating characteristic curve. rVSVΔG-ZEBOV-GP=recombinant vesicular stomatitis virus vector expressing the Zaire Ebola virus glycoprotein.

To circumvent this apparent limitation in cross-validating signatures identified in single cohorts, we next pooled the datasets of all four cohorts; to generate a balanced pooled dataset, we randomly down-sampled the Geneva and USA datasets. The resulting 10-gene signature (*BCL2*, *GBP5*, *IFI44*, *IFI44L*, *IFITM3*, *NLRP13*, *OAS2*, *OAS3*, *PRF1*, and *RAB33A*) exhibited excellent discriminatory values (AUC=94·8%) and excellent classifying capabilities in the balanced pooled cohort and in each single cohort (figure 5; appendix 6).

**Figure 6:**
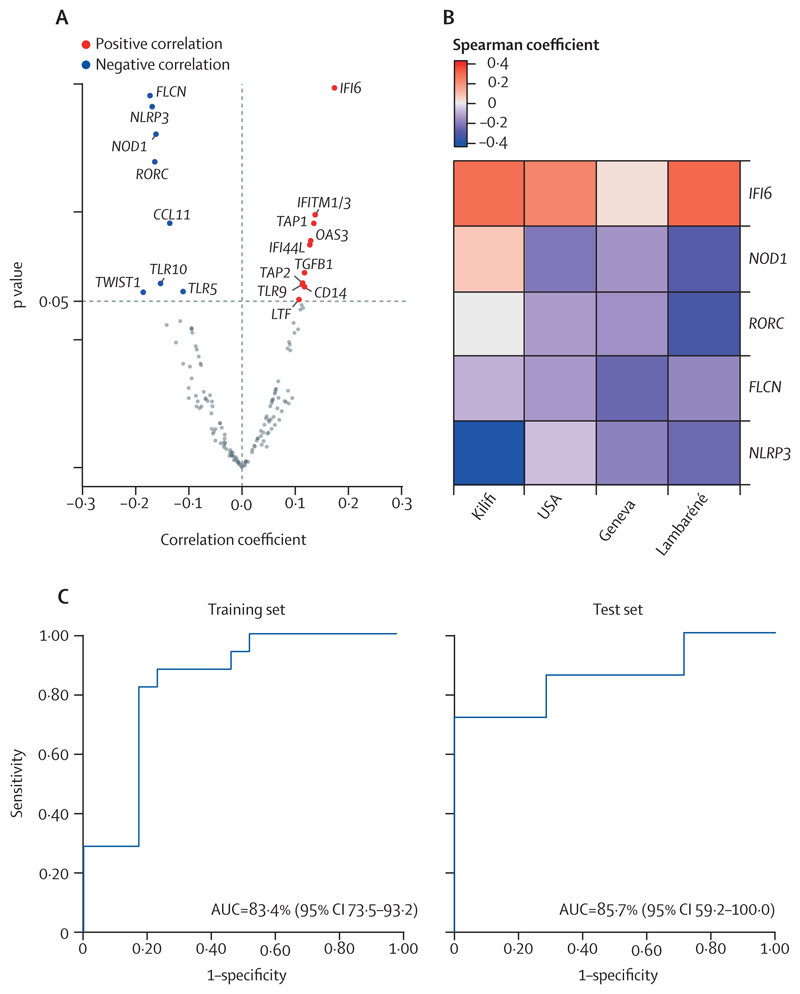
Correlation between gene expression profiles and ZEBOV-GP-specific antibody titres in response to rVSVΔG-ZEBOV-GP vaccination in cohorts from Geneva, USA, Lambaréné, and Kilifi and reactogenicity (arthritis) in the Geneva cohort (A) Volcano plot showing genes that significantly correlate with ZEBOV-GP-specific antibody titres in all cohorts using random-effects meta-analyses (p<0·05). (B) Heatmap showing the Spearman’s rank correlation coefficient of the genes included in the five-gene signature (p<0·05) that correlates with the ZEBOV-GP-specific antibody titres. (C) The receiver operating characteristic curves show the predictive power of the identified five-gene signature to classify participants at day 1 into those that will and those that will not develop arthritis in training and test set using the regularised logistic regression model. The curves display the values extracted from the regularised logistic regression model after hyperparameters adjustments. AUC=area under the receiver operating characteristic curve. *IFITM_1/3_*=*IFITM_1_* or *IFITM_3_*, or both. rVSVΔG-ZEBOV-GP=recombinant vesicular stomatitis virus vector expressing the Zaire Ebola virus glycoprotein. ZEBOV-GP=Zaire Ebola virus glycoprotein.

To identify early molecular correlates of immunogenicity shared between the cohorts, which is key for clinical application, Spearman correlations were calculated in each cohort between gene expression levels at day 7 after vaccination and ZEBOV-GP-specific IgG titres at days 28–30 after vaccination. Because this approach did not identify common gene signatures associated with immunogenicity, we next performed meta-analyses using random-effects models. Although the correlation coefficient was low (between –0·2 and 0·2), we were able to identify 18 genes whose expression significantly correlated with ZEBOV-GP-specific antibody responses in all four cohorts (figure 6A). The top five genes that had the smallest p value encompassed ISG *IFI6*, which positively correlated with antibody production and *FLCN*, *NLRP3*, *NOD1*, and *RORC* which negatively correlated with antibody production (figures 6A–B; appendix 2 p 29; appendix 9).

Finally, we aimed to identify genes that predict arthritis in the Geneva cohort at early timepoints, using recursive feature elimination from the entire day-1 dataset. The top five ranked genes used for machine learning model implementation were *CD4*, *CCR7*, *IL12A*, *FCGR1A*, and *GATA3* (appendix 2 p 29; appendix 9). To find the best machine learning predictive algorithm that could classify vaccinees into those who did or did not develop arthritis, several machine learning algorithms were evaluated. The regularised logistic regression model displayed superior performance in the training set (accuracy=79·4%, sensitivity=0·80, specificity=0·78, AUC=83·4% [95% CI 73·5–93·2]; figure 6C). We then evaluated our predictive model on the test set and our adjusted logistic regression model displayed a comparable performance (accuracy=86·0%, sensitivity=0·71, specificity=1·00, AUC=85·7% [59·2–100·0]; figure 6C).

## Discussion

Targeted gene expression profiling in adult healthy volunteers from Europe, North America, and Africa vaccinated with different doses of the rVSVΔG-ZEBOV-GP vaccine, identified (1) vaccine-related transcriptomic signatures, (2) a five-gene signature at day 7 after vaccination shared between all cohorts and associated with immunogenicity (ZEBOV-GP antibody titres), and (3) a five-gene signature at day 1 after vaccination in the Geneva cohort associated with development of arthritis. Our findings showed that the transcriptional response to vaccination is dependent on dose and time. Although we observed a prompt peak of gene expression perturbation at day 1 after vaccination, longitudinal transcriptomic profiling in rVSVΔG-ZEBOV-GP-vaccinated non-human primates showed a peak in the transcriptomic response at day 7 after vaccination, indicating that the vaccine response occurs with slower kinetics in non-human primates than in humans. Notwithstanding, DEGs identified at the peak of the response in both humans and non-human primates were dominated by shared ISGs and innate immunity-associated genes, suggesting a similar mode of action.^21^ Other studies reported the involvement of innate immunity following rVSVΔG-ZEBOV-GP vaccination^22^ as well as after the live-attenuated yellow fever vaccination YF-17D.^23^ Several slow-kinetics ISGs common in all four study cohorts (*IFI44L*, *OAS2, OAS3*, *IFIT2,* and *IFIT3*), have been shown to block viruses at the level of translation, replication, or both,^24^ potentially contributing to the rapidly induced post-vaccination protection in humans and non-human primates, even after exposure. Furthermore, IFN-inducible transmembrane family members (ie, *IFITM1* or *IFITM3*, or both) are known to block viral entrance and to inhibit early life-cycle steps of several viruses, including vesicular stomatitis virus, Ebola virus, and Marburg virus.^25,26^ Fast-kinetics ISGs (ie, *GBP1, GBP2,* and *GBP5*) are known to mediate host defence against different pathogens, including viruses,^27^ whereas *CXCL10* was observed to be upregulated not only after rVSVΔG-ZEBOV-GP vaccination (both at the transcriptional and protein level),^22^ but also after yellow fever^28^ and influenza^29^ vaccination. CXCL10 is known to stimulate the activation and migration of immune cells, such as monocytes, NK-cells, and T cells to the site of infection.^30^ Corroborating these data, NK markers (*NCAM1*), T-cell subsets markers (ie, *CD8A*, *CD3E*, *IL7R*, and *CD4*), and cytotoxicity markers (ie, *GNLY* and *GZMA*) were all downregulated in blood at the peak of the response, probably reflecting lymphocyte migration or marginalisation out of peripheral blood, as previously reported,^18,19,31^ whereas myeloid-associated genes (ie, *CD14* and *CD163*) were transiently upregulated.

Studies in animal models, including rodents and non-human primates, have underscored the importance of humoral immunity in protection from Ebola virus disease. ZEBOV-GP-specific total IgG levels have been shown to correlate with protection against a lethal ZEBOV dose.^32–34^ In humans, long-term persistence of ZEBOV-GP-specific IgG antibody responses (at least 1–2 years) following a single injection of the rVSVΔG-ZEBOV-GP vaccine has been reported,^35^ but early transcriptomic signatures correlating with immunogenicity have not yet been defined. We identified a five-gene signature shared between all cohorts that significantly correlated with immunogenicity of the rVSVΔG-ZEBOV-GP vaccine. Interestingly, only the IFN-inducible gene *IFI6* correlated positively with ZEBOV-GP-specific antibody titres. Consistent with our results, enrichment of IFN-signalling transcripts correlated with antibody titres after vaccination against influenza,^29,36^ malaria,^37^ dengue,^38^ rubella,^39^ and Ebola.^22^ These results might be explained by the fact that IFNα and IFNβ, normally activated during infections or immunisations, also execute immunoregulatory activities, being able to modify the adaptive immune system by direct T-cell and B-cell activation, thereby enhancing the production of specific antibodies.^40^ Alternatively, it was recently shown that *IFI6* reduced replication and transcription of modified Ebola virus,^41^ indicating that vaccine-induced expression of *IFI6* might have direct protective effects in addition to its correlation with antibody responses. Other innate immune response genes were among the genes that negatively correlated with the antibody response (ie, *FLCN*, *NLRP3,* and *NOD1*). A significant correlation between transcriptional apoptosis or survival, inflammasome markers, and pattern-recognition receptors with the humoral response was reported in two studies investigating the immune response following vaccination against hepatitis Band influenza.^29,42^ Characterising an early gene signature that might predict arthritis after rVSVΔG-ZEBOV-GP vaccine was one of our aims, given also the occurrence of arthritis following other vaccinations such as the measles, mumps, and rubella vaccination^43^ and Lyme disease vaccination.^44^ The five-gene signature included T-cell subset genes *CD4* and *CCR7*, IFN-signalling gene *FCGR1A*, myeloid-associated gene *IL12A*, and Th2-associated gene *GATA3*.

Previously, Huttner and colleagues^14^ identified a plasma signature that correlated with several adverse events, including arthritis, after rVSVΔG-ZEBOV-GP vaccination. Interestingly, several studies focussing on arthritis pathogenesis revealed that CD4^+^ T cells together with inflammatory and innate immune responses play a central role. In a mouse model for rheumatoid arthritis, CCR7^+^ CD4^+^ T cells accumulated and homed to lymphoid organs where they survived and maintained autoreactivity. Also, CD4^+^ T cell migration to peripheral tissues and their polarisation into Th1-cells was reported to be orchestrated by the proinflammatory cytokine IL-12.^45,46^ Moreover, inflammatory pathways are characterised by involvement of immunoregulatory genes, including *FCGR1A*.^47^ Although the classical pathways described in rheumatoid arthritis promote the differentiation of CD4^+^ T cells into Th1-cells, expression of Th2-cell marker *GATA3* might protect against severe joint inflammation by inhibiting differentiation of Th17-cells, but not Th1-cells, as reported in mice.^48^

Our study has limitations. In the African cohorts, we probably did not capture the peak of the response because the earliest samples for gene expression profiling were collected at day 7 after vaccination, which might have affected the overall comparisons between the African, European, and North American cohorts. Furthermore, differences in the sizes of the cohorts might have penalised the smaller cohorts in identifying robust gene predictors that discriminate among the classes (vaccinees before versus after vaccination). Additionally, we employed a high-throughput targeted gene expression profiling technique (dcRT-MLPA) using preselected immune-associated markers. Although this technique allowed profiling of large numbers of samples and proved successful, signatures might be refined in the future with more global techniques.

In summary, we have profiled and cross-validated the immune response-related, immunogenicity-related, and reactogenicity-related transcriptomic biomarker signatures induced by rVSVΔG-ZEBOV-GP vaccination in healthy adult participants. Our findings are novel, as they expand the analysis to four cohorts from different genetic and geographical backgrounds and report early shared transcriptomic signatures of immunogenicity and adverse events in response to Ebola vaccination. This study provides valuable insights into biological mechanisms that probably underlie the extremely high protective efficacy of rVSVΔG-ZEBOV-GP vaccination. Integrating additional high level omics data generated by different disciplines (ie, transcriptomics, metabolomics, and proteomics) will be required to further unravel the immune mechanisms responsible for early protection and reactogenicity following a single vaccination, enabling early interventions. Additionally, correlating early gene expression profiles with ZEBOV-GP IgG subclasses, antibody affinity, and neutralising titres in addition to total IgG might further improve the performance of immunogenicity-associated signatures, aiding in the rational design of new vaccines.

## Supplementary Material

Supplementary Material

## Data Availability

Data (eg, research data, R codes, and study protocols) are available upon reasonable request by e-mail directed to the corresponding author at e.vianello@lumc.nl and lead investigators of the clinical trials at claire-anne.siegrist@unige.ch (Geneva, Switzerland), agnandjis@cermel.org (Lambaréné, Gabon), and FNdungu@kemri-wellcome.org (Kilifi, Kenya). For the USA trial, the data sharing policy, including restrictions, of Merck Sharp & Dohme, a subsidiary of Merck, is available online (https://www.msdprivacy.com/us/en/cross-border-privacy-policy-rules/ and https://www.merckgroup.com/en/research/our-approach-to-research-and-development/healthcare/clinical-trials/commitment-responsible-data-sharing.html) and requests for access to the clinical study data can be submitted through the EngageZone site (https://engagezone.msd.com/ds_documentation.php) or via e-mail to dataaccess@merck.com. Requests will be assessed for scientific rigor before being granted, and a material transfer agreement might be required.

## Research in context

### Evidence before this study

Ebola virus disease outbreaks have high mortality rates and can be difficult to contain, such as during the large epidemic in west Africa (2014–16) and the most recent outbreak in the Democratic Republic of the Congo, which is still ongoing. Global efforts to control Ebola virus disease culminated in the development of several vaccines to prevent spread of the disease. We searched ClinicalTrials.gov up to Feb 3, 2020, for studies including “Ebola” and “vaccine” as keywords. 68 clinical trials have been reported in ClinicalTrials.gov. The recombinant vesicular stomatitis virus vector expressing the Zaire Ebola virus glycoprotein vaccine (rVSVΔG-ZEBOV-GP) was considered the leading candidate showing very safe, fast, and robust protective immunogenic profiles after a single injection in a phase 3 ring vaccination trial in Guinea during an outbreak in 2015. The characteristics of rVSVΔG-ZEBOV-GP make it an excellent vaccine candidate for outbreak events. rVSVΔG-ZEBOV-GP was prequalified by WHO on Nov 12, 2019, and licensed by the European Medicines Agency on Jan 14, 2021, and the US Food and Drug Administration on Dec 19, 2019, and is now on the market (under the name of Ervebo). Immune responses generated by this vaccine have proven highly effective against disease in outbreak settings. However, few studies have explored the mechanisms underlying these responses.

### Added value of this study

Our study expanded on previous knowledge about the mode of action of the rVSVΔG-ZEBOV-GP vaccine and unravelled, at the transcriptional level, the longitudinal and dose-dependent kinetics of innate and adaptive immune response profiles following vaccination. We extended the analysis to four cohorts of adult healthy volunteers from different genetic and geographical backgrounds (Europe, North America, and Africa) aiming to discover early common biomarker signatures of transcriptional response. Importantly, we identified gene expression signatures associated with immunogenicity, which correlated with ZEBOV-GP antibody titres, and with reactogenicity, which correlated with an adverse event (arthritis).

### Implication of all the available evidence

Our findings provide further insights into the mode of action by which rVSVΔG-ZEBOV-GP vaccination is protective and efficacious. The integration of our results with additional high level omics data (ie, transcriptomics, metabolomics, and proteomics) will contribute to further understanding of the immune mechanisms responsible for early protection following a single vaccination, and thus facilitate the design of new vaccines.
