# Supplementary material for: Transcriptomic signatures induced by the Ebola virus vaccine rVSVΔG-ZEBOV-GP in adult cohorts in Europe, Africa, and North America: a molecular biomarker study
Source: Lancet Microbe. Author manuscript; Available in PMC 2022 Sep 7. (PMC7613316; doi:10.1016/S2666-5247(21)00235-4)
Supplement: Supplementary Material [file EMS152556-supplement-Supplementary_Material.zip › 1-s2.0-S2666524721002354-mmc2.pdf]

THE LANCET
Microbe

Supplementary appendix 2

This appendix formed part of the original submission and has been peer reviewed. We post it as supplied by the authors.

Supplement to: Vianello E, Gonzalez-Dias P, van Veen S, et al. Transcriptomic signatures induced by the Ebola virus vaccine rVSVΔG-ZEBOV-GP in adult cohorts in Europe, Africa, and North America: a molecular biomarker study. *Lancet Microbe* 2021; published online Dec 6. https://doi.org/10.1016/S2666-5247(21)00235-4.

Transcriptomic signatures induced by the Ebola virus vaccine rVSVΔG-ZEBOV-GP in adult cohorts in Europe, Africa, and North America: a molecular biomarker study

Eleonora Vianello PhD [a,*], Patricia Gonzalez-Dias Msc [b], Suzanne van Veen Bsc [a], Carmen G. Engele Bsc [a], Edwin Quinten Bsc [a], Thomas P. Monath MD [c], Prof Donata Medaglini PhD [d,e], VSV-EBOVAC and VSV-EBOPLUS Consortia, Francesco Santoro PhD [d], Angela Huttner MD [f,g], Sheri Dubey PhD [h], Michael Eichberg PhD [h], Francis M. Ndungu PhD [i], Prof Peter G. Kremsner MD [j,k], Paulin N. Essone PhD [j], Selidji Todagbe Agnandji MD [j,k], Prof Claire-Anne Siegrist MD [f,g], Helder I. Nakaya PhD [b,l], Prof Tom H. M. Ottenhoff MD [a,†], and Mariëlle C. Haks PhD [a,†].

[a]*Department of Infectious Diseases, Leiden University Medical Center, Leiden, The Netherlands.*

[b]*Department of Clinical and Toxicological Analyses, School of Pharmaceutical Sciences, University of São Paulo, São Paulo, Brazil.*

[c]*NewLink Genetics Corp., Devens, MA 01439, USA.*

[d]*Laboratory of Molecular Microbiology and Biotechnology, Department of Medical Biotechnologies, University of Siena, Siena, Italy.*

[e]*Sclavo Vaccines Association, Siena, Italy.*

[f]*Division of Infectious Diseases, Geneva University Hospitals and Faculty of Medicine, Geneva, Switzerland.*

[g]*Center for Vaccinology, Geneva University Hospitals and Faculty of Medicine, Geneva, Switzerland.*

[h]*Department of Vaccine and Biologics Research, Merck Research Laboratories, West Point, PA 19486, USA.*

[i]*Department of Biosciences, KEMRI/Wellcome Trust Research Programme, Kilifi, 80108, Kenya.*

[j]*Centre de Recherches Médicales de Lambaréné, Lambaréné, Gabon.*

[k]*Institut für Tropenmedizin, Universitätsklinikum Tübingen, and German Center for Infection Research, Tübingen, Germany.*

[l]*Scientific Platform Pasteur-USP, São Paulo, Brazil.*

[†] Contributed equally

* Corresponding author: Dr. Eleonora Vianello
Department of Infectious Diseases
Leiden University Medical Center
Albinusdreef 2
2333 ZA Leiden
The Netherlands
Phone: +31 (0)71 526 3844
E-mail: e.vianello@lumc.nl

Table of contents

Supplementary Appendices **Page**

Appendix 1. Study cohort characteristics.

Table S1A: Details of the study design, setting and participant groups.

Table S1B: Baseline characteristics of the enrolled participants.

Appendix 2. Additional methods and supplementary figures

2.1	Ethical approval.	4
2.2	Detailed description of the dcRT-MLPA assay.	4
2.3	Detailed description of data processing and statistical analysis.	4
	Ingenuity Pathway Analysis (IPA)	4
	Principal Component Analysis (PCA)	4
	Molecular Degree of Perturbation (MDP)	4
	Logistic regression with lasso regularization	5
	Predicted probability plots: Visualizing the effects of logistic regression	5
	F1-score	5
	ELISA assay to detect ZEBOV glycoprotein-specific antibodies – details from previous studies	5
	Spearman correlation and random-effects models	5
	Identification of transcriptional signature associated with reactogenicity (arthritis) – Rationale for performing the analysis on Geneva cohort	6
	Recursive Feature Elimination (RFE) and machine learning (ML) algorithms	6

2.4	Figure S1:	PCA for the impact of dose on gene expression profiles following rVSVΔG-ZEBOV-GP vaccination in the Geneva cohort.	7
2.5	Figure S2:	Identification of differentially expressed genes (DEGs) following rVSVΔG-ZEBOV-GP vaccination in the Geneva cohort comparing HD_2 vs. LD.	8
2.6	Figure S3:	Identification of differentially expressed genes (DEGs) following rVSVΔG-ZEBOV-GP vaccination in the Geneva and USA cohorts comparing vaccinees vs. placebo controls.	9
2.7	Figure S4:	Expression kinetics of representative genes in the Geneva cohort following rVSVΔG-ZEBOV vaccination.	10
2.8	Figure S5:	IPA analysis of the DEGs identified in the Geneva cohort at d1 following rVSVΔG-ZEBOV-GP vaccination in HD_2 vaccinees compared to LD vaccinees.	12
2.9	Figure S6:	Impact of time and dose on gene expression profiles following rVSVΔG-ZEBOV-GP vaccination in the USA cohort.	13
2.10	Figure S7:	Identification of differentially expressed genes (DEGs) and key networks following rVSVΔG-ZEBOV-GP vaccination in the USA cohort.	14
2.11	Figure S8:	Expression kinetics of representative genes in the USA cohort following rVSVΔG-ZEBOV-GP vaccination.	16
2.12	Figure S9:	Venn diagram depicting DEGs shared between the different study cohorts.	18
2.13	Figure S10:	Impact of time and dose on gene expression profiles and identification of DEGs and key networks following rVSVΔG-ZEBOV-GP vaccination in the Lambaréné cohort.	19
2.14	Figure S11:	Expression kinetics of representative genes in the Lambaréné cohort following rVSVΔG-ZEBOV-GP vaccination.	21
2.15	Figure S12:	Impact of time and dose on gene expression profiles and identification of DEGs and key networks following rVSVΔG-ZEBOV-GP vaccination in the Kilifi cohort.	23
2.16	Figure S13:	Expression kinetics of representative genes in the Kilifi cohort following rVSVΔG-ZEBOV-GP vaccination.	25
2.17	Figure S14:	Identification of signatures associated with rVSVΔG-ZEBOV-GP vaccination at the peak of the transcriptomic response (d1) in the Geneva and USA cohorts.	27
2.18	Figure S15:	Identification of common signatures associated with rVSVΔG-ZEBOV-GP vaccination at d7 post-vaccination in all 4 study cohorts.	28
2.19	Figure S16:	Correlation between gene expression profiles and ZEBOV-GP-specific antibody titers in response to rVSVΔG-ZEBOV-GP vaccination in cohorts from Europe, USA, and Africa and reactogenicity (arthritis) in the Geneva cohort.	29

Appendix 3. Table S3: List of target genes for dcRT-MLPA.
Appendix 4. Table S4: dcRT-MLPA gene expression data (normalized to *GAPDH* and $\log_2$-transformed).
Table S4A: dcRT-MLPA gene expression data of the Geneva (Switzerland) cohort.
Table S4B: dcRT-MLPA gene expression data of the USA cohort.
Table S4C: dcRT-MLPA gene expression data of the Lambaréné (Gabon) cohort.
Table S4D: dcRT-MLPA gene expression data of the Kilifi (Kenya) cohort.
Appendix 5. Table S5: Differential Expression Analysis (Mann-Whitney test with Benjamini-Hochberg correction for multiple testing, p <0.05, $\log_2$ FC ≤-0·6 and ≥0·6).
Table S5A: Differential Gene Expression Analysis in Geneva cohort, all vaccinees vs. Day 0.
Table S5B: Differential Gene Expression Analysis in Geneva cohort, HD_2 (1-5x10^7 pfu) vs. LD (3x10^5 pfu).
Table S5C: Differential Gene Expression Analysis in Geneva cohort, all vaccinees vs. placebo.
Table S5D: Differential Gene Expression Analysis in Geneva cohort, HD_2 (1-5x10^7 pfu) vs. Day 0.
Table S5E: Differential Gene Expression Analysis in Geneva cohort, LD (3x10^5 pfu) vs. Day 0.
Table S5F: Differential Gene Expression Analysis in USA cohort, all vaccinees vs. Day 0.
Table S5G: Differential Gene Expression Analysis in USA cohort, HD_2 (2x10^7 pfu) vs. ID (3x10^6 pfu).
Table S5H: Differential Gene Expression Analysis in USA cohort, all vaccinees vs. placebo.
Table S5I: Differential Gene Expression Analysis in USA cohort, HD_2 (2x10^7 pfu) vs. Day 0.
Table S5J: Differential Gene Expression Analysis in USA cohort, ID (3x10^6 pfu) vs. Day 0.
Table S5K: Differential Gene Expression Analysis in Lambaréné cohort, all vaccinees vs. Day 0.
Table S5L: Differential Gene Expression Analysis in Lambaréné cohort, HD_2 (2x10^7 pfu) vs. ID (3x10^6 pfu).
Table S5M: Differential Gene Expression Analysis in Lambaréné cohort, HD_2 (2x10^7 pfu) vs. LD (3x10^5 pfu).
Table S5N: Differential Gene Expression Analysis in Lambaréné cohort, ID (3x10^6 pfu) vs. LD (3x10^5 pfu).
Table S5O: Differential Gene Expression Analysis in Lambaréné cohort, HD_2 (2x10^7 pfu) vs. Day 0.
Table S5P: Differential Gene Expression Analysis in Lambaréné cohort, ID (3x10^6 pfu) vs. Day 0.
Table S5Q: Differential Gene Expression Analysis in Lambaréné cohort, LD (3x10^5 pfu) vs. Day 0.
Table S5R: Differential Gene Expression Analysis in Kilifi cohort, all vaccinees vs. Day 0.
Table S5S: Differential Gene Expression Analysis in Kilifi cohort, HD_2 (2x10^7 pfu) vs. ID (3x10^6 pfu).
Table S5T: Differential Gene Expression Analysis in Kilifi cohort, HD_2 (2x10^7 pfu) vs. Day 0.
Table S5U: Differential Gene Expression Analysis in Kilifi cohort, ID (3x10^6 pfu) vs. Day 0.
Appendix 6. Table S6: Identification of signatures associated with vaccination.
Table S6A: Signatures associated with vaccination: Day 1 vs Day 0.
Table S6B: Signatures associated with vaccination: Day 7 vs Day 0.
Appendix 7. Table S7: Statistical analyses (one-tailed unpaired T-test) corresponding to predicted probability plots.
Table S7A: Statistical analyses corresponding to predicted probability plots: Day 1 vs Day 0.
Table S7B: Statistical analyses corresponding to predicted probability plots: Day 7 vs Day 0.
Appendix 8. Table S8: F1-scores from the logistic regression with lasso regularization model for Training-Test and Validation cohorts: Day 1 and Day 7.
Appendix 9. Table S9: Signatures that correlate with ZEBOV-GP specific IgG titers or with the development of arthritis.
Appendix 10. Supplementary References 30

Appendixes 1, 3, 4, 5, 6, 7, 8, and 9 are available online as a downloadable Microsoft Excel document.

Appendix 2. Additional methods and supplementary figures

2.1 Ethical approval

The trial protocols were reviewed and approved by the WHO's Ethics Committee as well as by local ethics committees (Geneva trial: the Geneva Cantonal Ethics Commission and the Swiss Agency for Therapeutic Products (Swissmedic); USA trial: the Chesapeake Institutional Review Boards (Columbia, MD, USA) and the Crescent City Institutional Review Board (New Orleans, LA, USA); Lambaréné trial: the Scientific Review Committee of Centre de Recherches Médicales de Lambaréné (CERMEL), the Institutional Ethics Committee of CERMEL, the National Ethics Committee of Gabon, and the Institutional Ethics Committee of the Universitätsklinikum Tübingen; Kilifi trial: Kilifi Ethics Committee).

2.2 Detailed description of the dual-color Reverse-Transcriptase Multiplex Ligation-dependent Probe Amplification (dcRT-MLPA) assay

For each target-specific sequence, a specific RT primer was designed located immediately downstream of the left- and right-hand half-probe target sequence. RNA was reverse transcribed to cDNA by incubating at 37°C for 15 minutes, using RT-primer mix and the Moloney Murine Leukemia Virus (M-MLV) reverse transcriptase kit (Promega, Leiden, The Netherlands). The reverse transcriptase enzyme was inactivated by heating at 98°C for 2 minutes.

The left- and right-hand half probes were hybridized to the cDNA at 60°C overnight and annealed half-probes were ligated at 54°C for 15 minutes using ligase-65. Ligase-65 was subsequently inactivated by heating at 98°C for 5 minutes. Ligated probes were amplified by PCR (33 cycles at 95°C for 30 seconds, 58°C for 30 seconds and 72°C for 60 seconds, followed by one cycle at 72°C for 20 minutes). To monitor assay performance, substituting RNA with nuclease-free water (ThermoFisher Scientific, Wilmington, DE, USA) was used as negative control while Human Universal References RNA (Clontech, Palo Alto, CA, USA) and synthetic template oligonucleotides as hybridization templates were used as positive controls. Primers and probes were from Sigma-Aldrich Chemie (Zwijndrecht, The Netherlands) and the SALSA MLPA reagent kit from MRC-Holland (Amsterdam, The Netherlands). RT primers and half-probes were designed by Leiden University Medical Centre (LUMC, Leiden, The Netherlands)[1,2] and comprised sequences for 4 housekeeping genes and 144 selected key immune-related genes to profile the following compartments of the human immune response (Table S2): (1) Adaptive immune responses: T-cell responses; Th1 responses; Th2 responses; Th17/22 responses; Treg responses; T-cell cytotoxicity; Immune cell subset markers including B-cells and NK-cells. (2) Innate immune responses: Myeloid-associated markers and scavenger receptors; Pattern recognition receptors; Inflammasome components. (3) Inflammatory and IFN-signalling genes. (4) Other genes: Anti-microbial activity; Apoptosis/cell survival; E3 ubiquitin protein ligases; Small GTPases/(Rho)GTPase activating proteins; Additional chemokines; Cell growth/proliferation; Cell activation; Transcriptional regulators/activators; Intracellular transport; Mitochondrial Stress/Proteasome; Inflammation.

PCR products were 1:10 diluted in Highly deionized (Hi-Di) formamide (ThermoFisher) containing 400HD Rhodamine X (ROX) fluorophore size standard (ThermoFisher). PCR products were denatured at 95°C for 5 min, stored immediately at 4°C and analyzed on an Applied Biosystems 3730 capillary sequencer in GeneScan mode (BaseClear, Leiden, The Netherlands).

Trace data were analyzed using GeneMapper software 5 (Applied Biosystems, Warrington, UK). The areas of each assigned peak (arbitrary units) were exported for analysis in R (version 3.5.1). Data were normalized to housekeeping gene glyceraldehyde 3-phosphate dehydrogenase (GAPDH) and signals below the threshold value for noise cutoff in GeneMapper ($\log_2$ transformed peak area 7·64) were assigned the threshold value for noise cutoff. Finally, the normalized data were $\log_2$-transformed for statistical analysis.

2.3 Detailed description of data processing and statistical analysis

Ingenuity Pathway Analysis (IPA)

IPA (IPA-60467501) (QIAGEN, Hilden, Germany) was used to explore networks and relationships between the DEGs identified in each cohort. The top scoring interactive network is shown. Genes are indicated by nodes that are displayed using various shapes that represent the functional class of the gene product. Relationships between the nodes are indicated by edges. All edges are supported by at least one reference from the literature, or from canonical information stored in the Ingenuity Pathways Knowledge Base. Solid edges represent direct protein-protein interactions while dashed edges represent indirect interactions.

Principal Component Analysis (PCA)

Principal Component Analysis (PCA) was used for dimensionality reduction to explore the dataset (function *prcomp* from the stats R package).[3] The data was projected onto the first two principal components to obtain lower-dimensional data while preserving the maximum data variation.

Molecular Degree of Perturbation (MDP)

The molecular Degree of Perturbation (MDP) is a package implemented in R (R package *mdp*)[4], which is based on the Molecular Distance to Health[5]. The MDP algorithm quantifies the heterogeneity of samples within a group using transcriptome data. In general terms, MDP calculates the degree of perturbation of each gene relative to the same gene identified in a control group of

samples. Only with those highly disturbed genes, a representative disturbance score is set for each sample. Therefore, MDP can identify so-called sound samples that present some alteration in the transcriptome unidentified by phenotype.

Essentially, the MDP score represents the differences by number of standard deviations in the control group. The formulas used to calculate the MDP in the present study are presented below:

$$Molecular\,inflammatory\,perturbation = \frac{x_i - \bar{x}_{(reference)}}{\sigma_{(reference)}}$$

$$\sigma = \frac{\sum_{(i=1)}^{n} (x_i - \bar{x})^2}{(n-1)}$$

ɳ = Number of data points
x_i = Each of the value of data
$\bar{x}$ = Mean of the data points
σ = Standard deviation

In this study, we applied the MDP scoring system using data from 144 genes quantified in four different cohorts of vaccinated volunteers and controls prior to vaccination. The MDP transformation was used as an approach to normalize cross-data experiments, resulting in data sets with markers distributed on a similar scale.

Logistic regression with lasso regularization

To identify host biomarker signatures with the best discriminatory capability, penalized logistic regression was used as a classification method. We performed logistic regression with lasso regularization to reduce the set of variables keeping the most relevant (the penalty in lasso forces some of the coefficient estimates with a minor contribution to the model to be exactly equal to zero) and improving the discrimination performance between the selected classes (specific timepoints). We used the R packages *glmnet*[6] to fit regression models with regularizations and *penalized* to compare and validate results with high dimensional penalized regression models.[7,8] Leave-One-Out Cross Validation (LOOCV) and Train-Test Split (TTS) (training set = 70% of the dataset, test set = 30% of the dataset) were used in both packages to assess the performance of the trained models. When datasets of cohorts that differed substantially in the number of subjects that were included were pooled, a random down-sampling approach using R was applied to obtain a balanced distribution of the number of subjects within the pooled cohort. Receiver Operating Characteristic (ROC) curves (Sensitivity plotted against 1-Specificity), Area Under the Curve (AUC) with 95% confidence intervals (CI), F1 scores (harmonic mean of precision and recall), and box-and-whiskers-plots representing the predicted probability for each class were used to evaluate the classifying performance of the trained models. Candidate signatures were extracted from the trained models using the non-zero coefficients of the logistic regression model selected by the lasso regularization. In order to provide a more robust signature we only considered genes that were identified by both *glmnet* and *penalized* packages. Plots were generated using the R package *ggplot2*.

Predicted probability plots: Visualizing the effects of logistic regression

The Predicted probability is the probability of an event that is calculated from available data. The application of penalized logistic regression as classification method to the dataset allows the calculation of the probability of the occurrence of a specific class, which is its predicted classification. In the datasets described in this manuscript, 2 classes were defined: 0, which is always equal to day 0 (not vaccinated) and 1, which can be any timepoint post-vaccination. The closer the calculated predicted probability is to 1 the more likely, according to the model, the 1 was the actual class (the volunteer was vaccinated). Vice versa the closer the calculated predicted probability is to 0, the more likely the 0 was the actual class (the volunteer was not vaccinated).

F1-score

The F1-score is the harmonic mean of Precision (the measure of the correctly identified positive cases from all the predicted positive cases) and Recall (the measure of the correctly identified positive cases from all the actual positive cases). The precision is an important metric when the costs of False Positives is high, while the recall is an important metric when the costs of False Negatives is high. The F1-score is the preferred measure for models with imbalanced class distribution (as in our study). In contrast, the accuracy is the measure of all the correctly identified cases and is mostly used when all the classes are equally important and in general when the classes are balanced.

ELISA assay to detect ZEBOV glycoprotein-specific antibodies – details from previous studies

ELISA assays to detect ZEBOV-GP-specific IgG antibody titers have been performed for previously published studies[9-11] and used in this study for the correlation analysis with log_2-transformed gene expression data (d7 post-vaccination). Briefly, the serum samples of the Geneva, Kilifi, and Lambaréné cohorts were transferred to the Non-Clinical Development laboratory at the US Army Medical Research Institute for Infectious Diseases (USAMRIID), Fort Detrick, MD, USA. ZEBOV glycoprotein-specific antibodies were quantified with the Filovirus Animal Non-Clinical Group (FANG)-approved ELISA by use of the homologous Zaire-Kikwit strain glycoprotein, following USAMRRIID's standard operating procedure (SOP AP-03-35; USAMRIID ELISA). The serum samples of the USA cohort were transferred to Focus Diagnostics, San Juan Capistrano, CA. The ELISA assay was based on the assay developed by the Filovirus Animal Non-Clinical Group (FANG).

Spearman correlation and random-effects models

To identify signatures associated with immunogenicity, pairwise log_2-transformed gene expression data (d7 post-vaccination) were correlated with ZEBOV-GP-specific IgG enzyme-linked immunosorbent assay antibody titers (determined at d28 post-

vaccination) using Spearman correlation in each cohort. Only significant correlations ($p<0{\cdot}05$) were considered. Effect sizes of gene expression and antibody titer correlation analyses obtained in single cohorts were subsequently integrated using the random-effects models for meta-analyses, as implemented in the *metafor* R package. Random-effects models have been chosen because of the heterogeneity of the data (different cohorts, timepoints, sample sizes), which is shown through a confidence interval. This interval assesses the amount of heterogeneity in a meta-analysis, allowing an evaluation of whether the grouping of the estimates is significant. For the correlation-based analyses, sampling variances of Spearman Correlation Coefficient (SCCs) from each cohort were estimated using the *escalc* function. To estimate the summarized effect sizes, SCCs and variances were submitted to the *rma* function using Hunter and Schmidt (HS). Studies were weighted by the inverse of variance.[12]

Identification of transcriptional signature associated with reactogenicity (arthritis) - Rationale for performing the analysis on Geneva cohort

The rVSVΔG-ZEBOV-GP vaccine was reported to be safe, protective, and immunogenic, but also reactogenic. Arthritis was a significant and debilitating adverse event described in 23·5% (24/102) of the volunteers in the Geneva cohort independent of dose.[9,11] The onset of arthritis occurred around d10-14 after injection and was characterized by a prolonged duration (more than a week). In contrast, only 4·5% (19/418) of the volunteers in the USA cohort developed arthritis [10] (for this study data of only 4 participants out of 125 were available (3.2% of arthritis incidence in USA considering these numbers)), and in the African cohorts only 1 case was reported (Kilifi cohort).[11] Since other adverse events observed in the 4 study cohorts, such as pain at the site of injection, fever, nausea, and fatigue were characterized by an early onset (d1/d2 post-vaccination) and were short lasting (24-48 hours), we focused our analysis on the identification of gene predictors of arthritis that could possibly be used in future studies to predict the occurrence of arthritis early after vaccination (d1) as well as help in developing new vaccines. Our analysis was limited to the Geneva cohort, because of the highest percentage of arthritis among vaccinated participants and gene expression data of all 24 participants affected by this adverse event were available. The lower incidence in the USA and Kilifi cohorts (4 and 1 samples, respectively) was insufficient to effectively predict arthritis separately in these cohorts. Moreover, combining samples from the three cohorts that recorded arthritis in a single dataset would likely result in more heterogeneous and less robust models. Since our dataset is already affected by unbalanced classes (our dataset encompasses lot more data from participants without arthritis than from participants affected with arthritis), we decided not to add more bias to the data, since the data from the other cohorts has dispersions different from the Geneva cohort.

Recursive Feature Elimination method (RFE) and machine learning (ML) algorithms

To identify signatures associated with reactogenicity (arthritis), volunteers from the Geneva cohort were split into training (70%) and test (30%) sets. To balance the dataset, we applied a down-sampling approach, reducing the number of subjects that did not develop arthritis to obtain an equal distribution with the number of subjects that did develop arthritis. This was necessary to prevent the model from tending to classify better the majority class (control = no arthritis) than the minority class (arthritis). The performance of the model and the reliability of the data have been verified to avoid unwanted bias. Then, the adjusted dataset was split into training set (70%) and test set (30%). Recursive Feature Elimination method (RFE)[13], available in the *caret* R package[14], was applied to the training set to select the top-ranking genes able to separate vaccinees that will develop arthritis from vaccinees that will not develop arthritis. RFE is a method of selecting features that fits a model and removes the weakest feature (or features) until the specified number of features is reached. Features are classified by the importance of the model's features and, by recursively eliminating a small number of features per loop, RFE tries to eliminate dependencies and collinearity that may exist in the model. To find the optimal number of features to maintain, cross-validation is used with RFE to score different subsets of features and select the best collection of feature scores. The result of the RFE shows the number of features in the model together with its cross-validation test score and variability. From this, the lowest number of features with the best performance is selected.

Once these top genes were identified, their expression values were extracted from both the training and test datasets. We subsequently trained different ML algorithms (regLogistic, dnn, CART, ada, rpart, nnet, plr, kNN, SVM, RF, bayesglm, adaboost and xgBoost) available on the *caret* R package on the training set and evaluated the training performance by cross-validation (5 k-fold). Regularized Logistic Regression (LR) model, tuned by adjusting some hyperparameters (loss = L1, cost of constraints violation (cost = 1), and tolerance of termination criterion for optimization (epsilon = 1)) was identified as the best algorithm. We tested the model on the test set and assessed the classifying performance of the model by evaluating sensitivity, specificity, Receiver Operating Characteristic (ROC) curve, and Area Under the ROC Curve (AUC) with 95% Confidence Interval (CI).

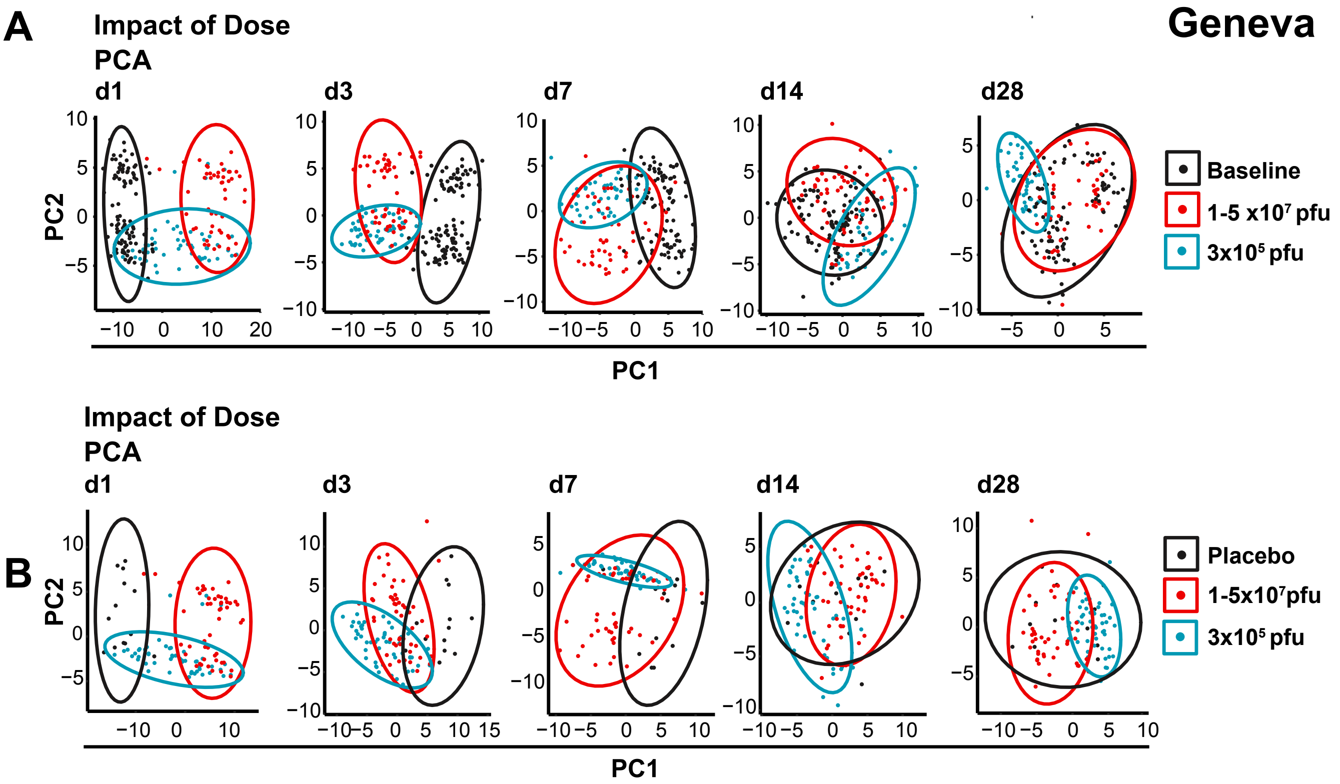

Figure S1. PCA for the impact of dose on gene expression profiles following rVSVΔG-ZEBOV-GP vaccination in the Geneva cohort.

(A) PCA analysis at distinct timepoints. Samples were separated per dose to evaluate the impact of the dose. d0 samples of vaccinees were used as baseline controls. (B) PCA analysis at distinct timepoints. Samples were separated per dose to evaluate the impact of the dose. Placebo vaccinated individuals were used as control group.

Geneva

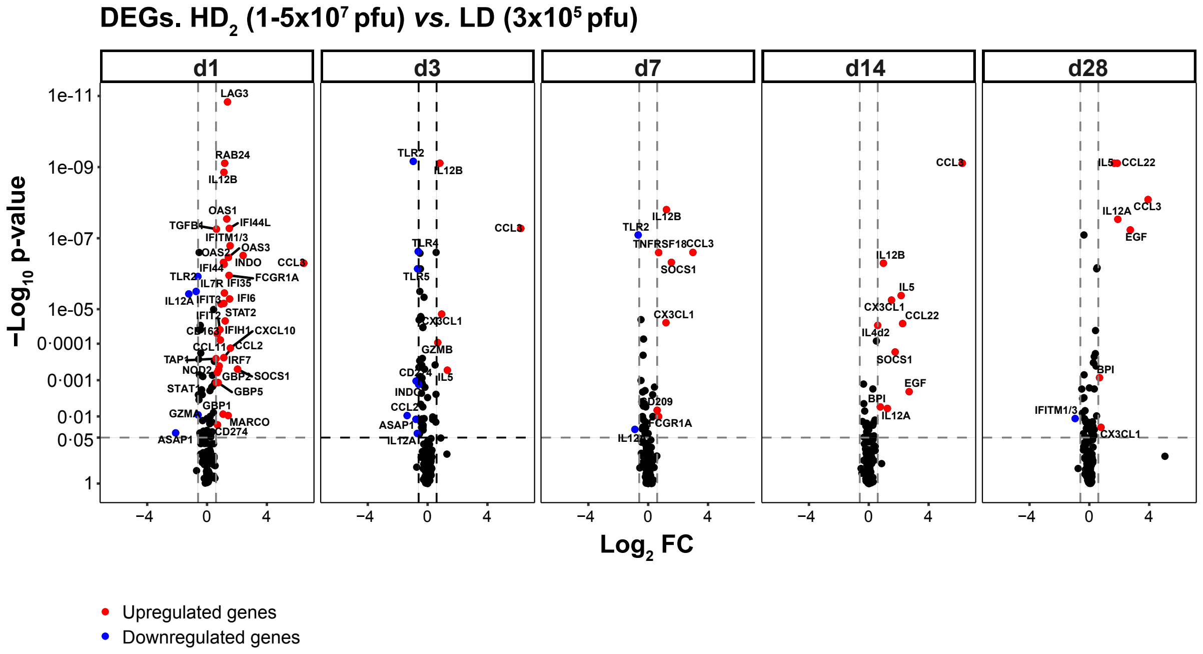

Figure S2. Identification of differentially expressed genes (DEGs) following rVSVΔG-ZEBOV-GP vaccination in the Geneva cohort comparing HD_2 versus LD.

Differential Expression Analysis was performed on GAPDH-normalized log_2-transformed gene expression data of the Geneva cohort. Volcano plots representing DEGs at different timepoints (d1, 3, 7, 14, and 28) following rVSVΔG-ZEBOV-GP vaccination comparing vaccinees that received a HD_2 (1-5x10^7 pfu) versus a LD (3x10^5 pfu). The y-axis scales of all plots are harmonized. P-values, -log_{10}-transformed for better visualization, are plotted against log_2 FC. Genes with p <0·05 and log_2 FC <-0·6 or >0·6 were labelled as DEGs

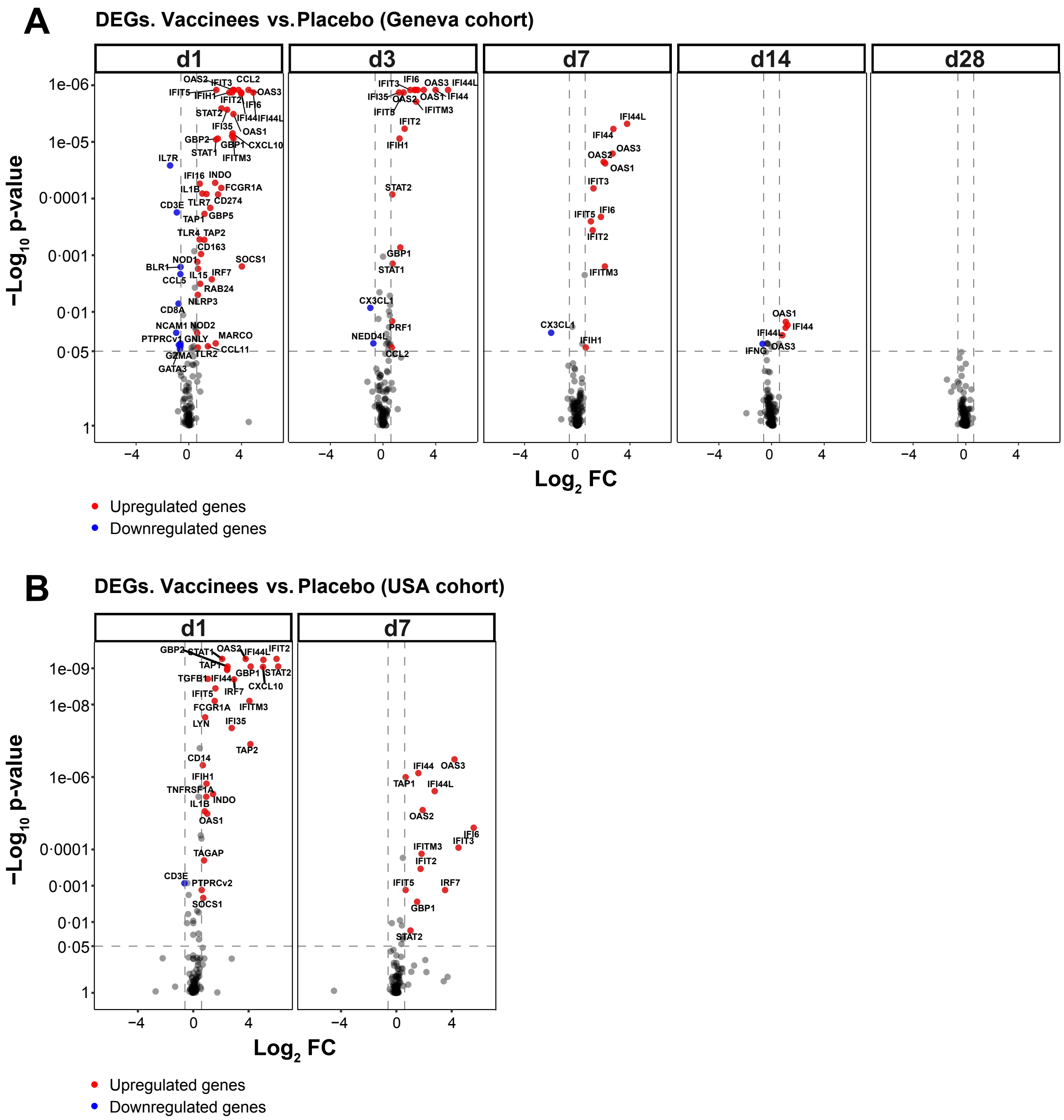

Figure S3. Identification of differentially expressed genes (DEGs) following rVSVΔG-ZEBOV-GP vaccination in the Geneva and USA cohorts comparing vaccinees vs. placebo controls. Differential Expression Analysis was performed on *GAPDH*-normalized log_2-transformed gene expression data of the Geneva and USA cohorts. (A) Volcano plots representing DEGs at different timepoints (d1, 3, 7, 14, and 28) following rVSVΔG-ZEBOV-GP vaccination of all vaccinees compared to the placebo controls in the Geneva cohort. The y-axis scales of all plots are harmonized. P-values, $-log_{10}$-transformed for better visualization, are plotted against log_2 FC. Genes with $p < 0·05$ and log_2 FC <-0·6 or >0·6 were labelled as DEGs. (B) Volcano plots representing DEGs at different timepoints (d1, 7) following rVSVΔG-ZEBOV-GP vaccination of all vaccinees compared to the placebo controls in the USA cohort. The y-axis scales of all plots are harmonized. P-values, $-log_{10}$-transformed for better visualization, are plotted against log_2 FC. Genes with $p < 0·05$ and log_2 FC <-0·6 or >0·6 were labelled as DEGs.

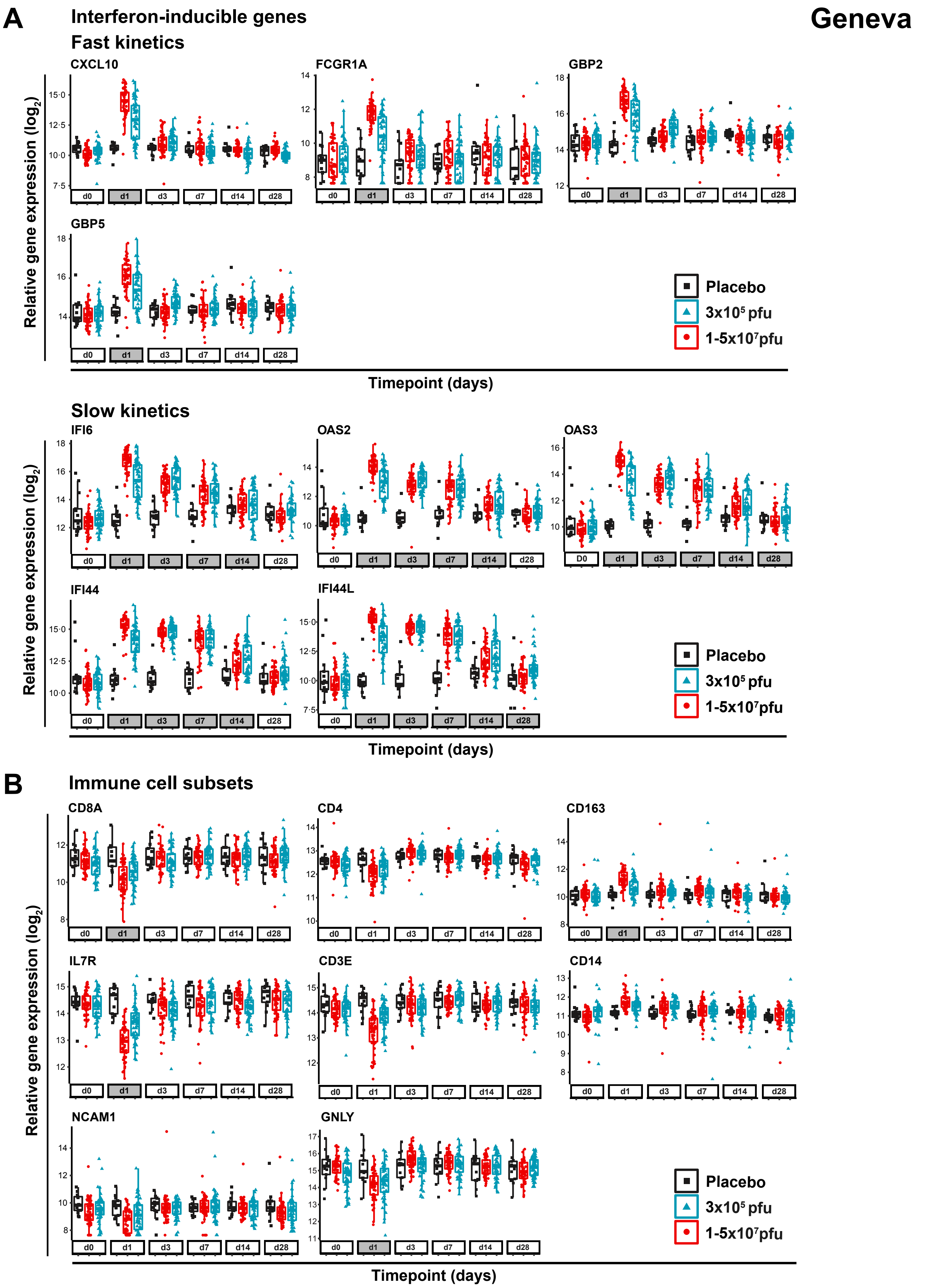
A
Interferon-inducible genes
Fast kinetics
Geneva
CXCL10
FCGR1A
GBP2
GBP5
Relative gene expression (log2)
Placebo
3x10⁵ pfu
1-5x10⁷pfu
Timepoint (days)
Slow kinetics
IFI6
OAS2
OAS3
IFI44
IFI44L
Relative gene expression (log2)
Placebo
3x10⁵ pfu
1-5x10⁷pfu
Timepoint (days)
B
Immune cell subsets
CD8A
CD4
CD163
IL7R
CD3E
CD14
NCAM1
GNLY
Relative gene expression (log2)
Placebo
3x10⁵ pfu
1-5x10⁷pfu
Timepoint (days)
d0
d1
d3
d7
d14
d28

Figure S4. Expression kinetics of representative genes in the Geneva cohort following rVSVΔG-ZEBOV-GP vaccination. Log_2-transformed relative transcript levels of (A) interferon-inducible genes displaying either fast (top panel) or slow (bottom panel) kinetics and (B) immune cells subset genes. Box plots depict median gene expression values and the inter quartile range (IQR), while the whiskers represent the data within the Q_1-1·5xIQR and Q_3+1·5xIQR interval. Outliers are reported as symbols. Vaccinees receiving either a HD_2 (1-5x10^7 pfu), LD (3x10^5 pfu), or placebo control (P) are represented by distinctive colors. Timepoints post-vaccination at which genes are differentially expressed compared to baseline are highlighted in grey. Statistical significance ($p < 0·05$) was determined using a non-parametric Mann-Whitney test with Benjamini-Hochberg correction for multiple testing.

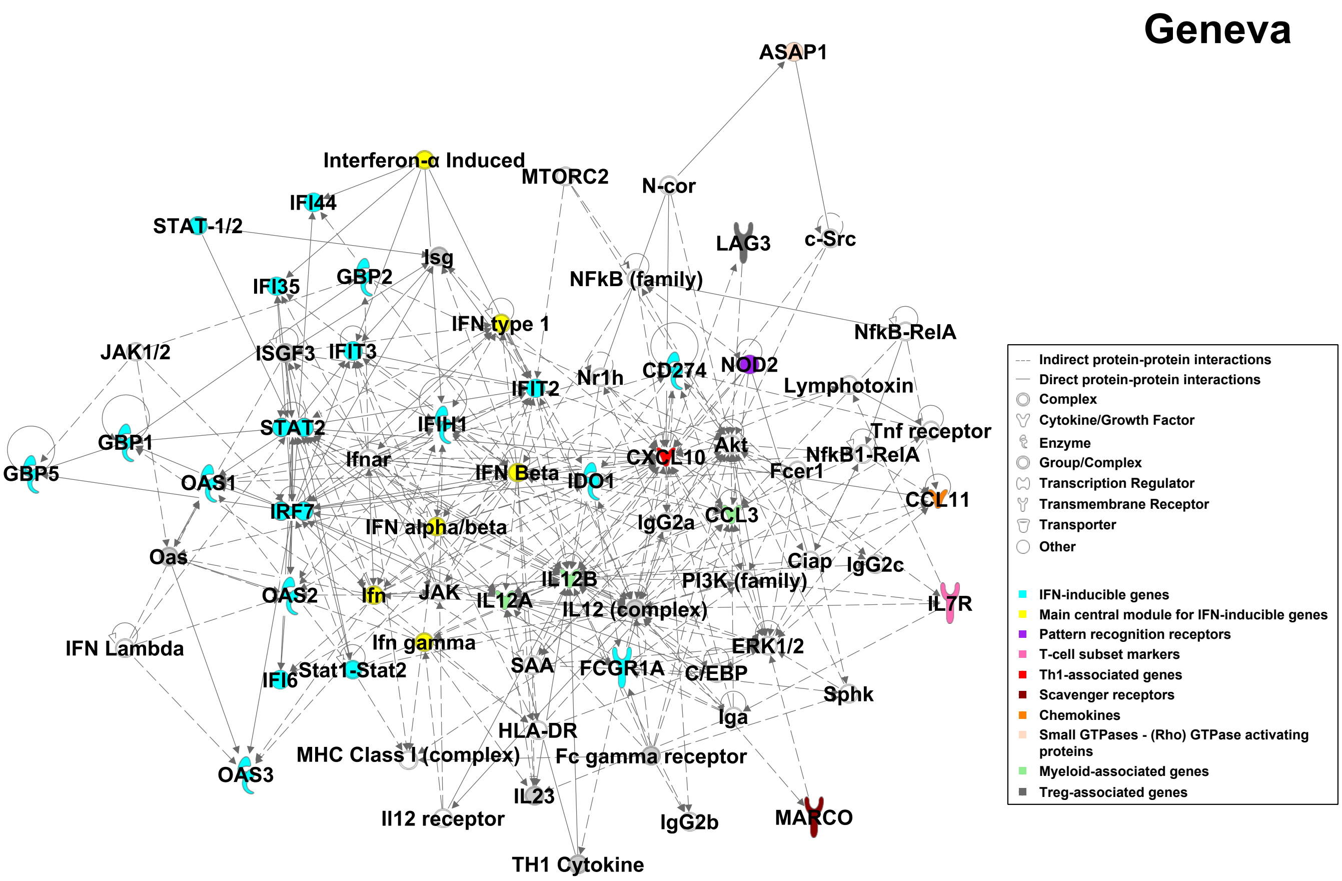

Figure S5. IPA analysis of the DEGs identified in the Geneva cohort at d1 following rVSVΔG-ZEBOV-GP vaccination in HD_2 vaccinees compared to LD vaccinees. The various shapes of the nodes represent the functional classes of the gene products.

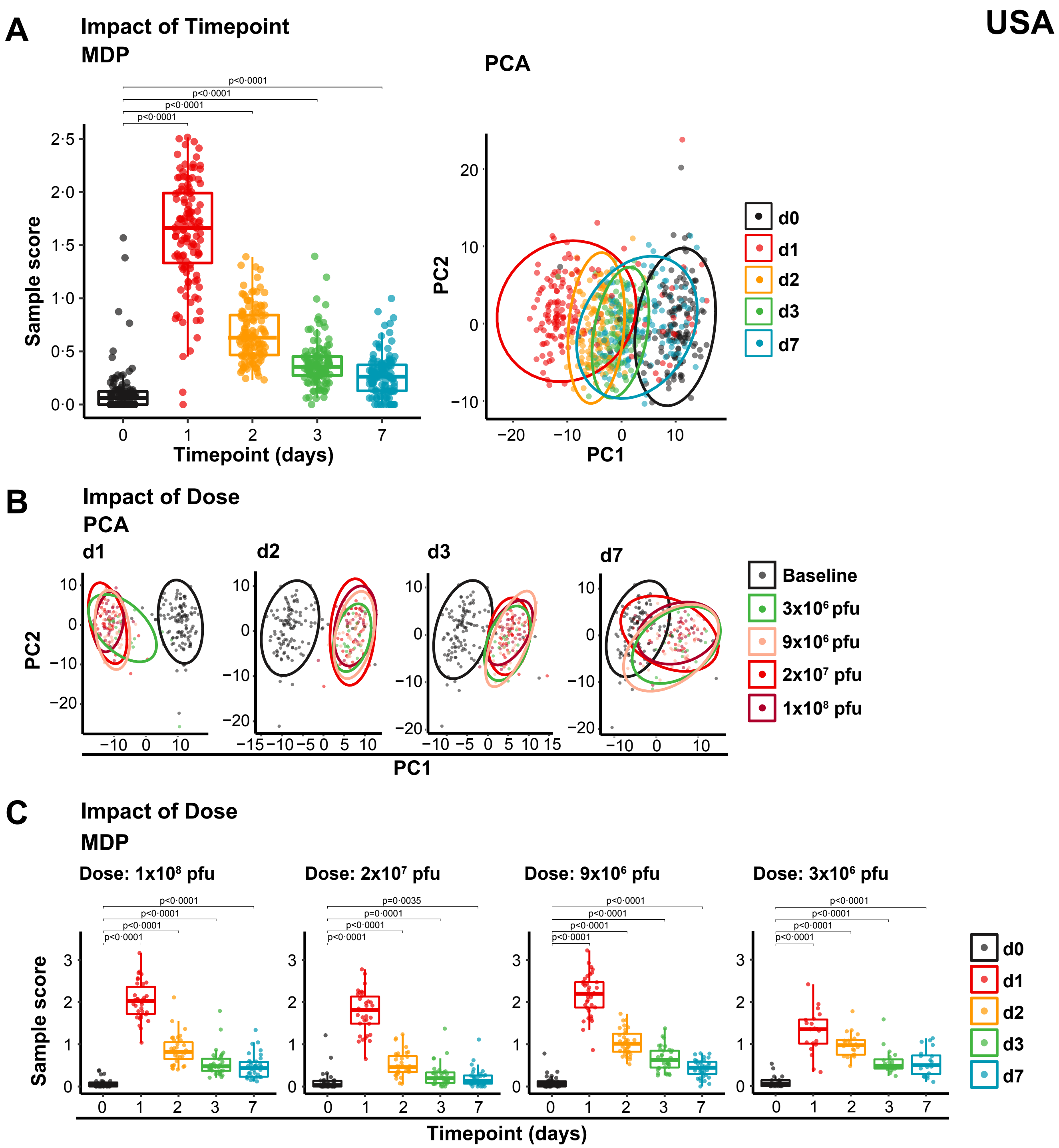

Figure S6. Impact of time and dose on gene expression profiles following rVSVΔG-ZEBOV-GP vaccination in the USA cohort. Molecular Degree of Perturbation (MDP) and Principal Component Analysis (PCA) were performed on GAPDH-normalized $\log_2$-transformed gene expression data of the USA cohort. (A) Samples were separated by timepoint (d0, 1, 2, 3, and 7) to evaluate the impact of time. d0 samples of vaccinees were used as baseline controls. Timepoints were compared using Mann-Whitney U test. (B) PCA analysis at distinct timepoints. Samples were separated per dose to evaluate the impact of the dose. d0 samples of vaccinees were used as baseline controls. (C) MDP analysis at distinct timepoints. Samples were separated per dose to evaluate the impact of the dose. d0 samples of vaccinees were used as baseline controls. Timepoints were compared using Mann-Whitney U test.

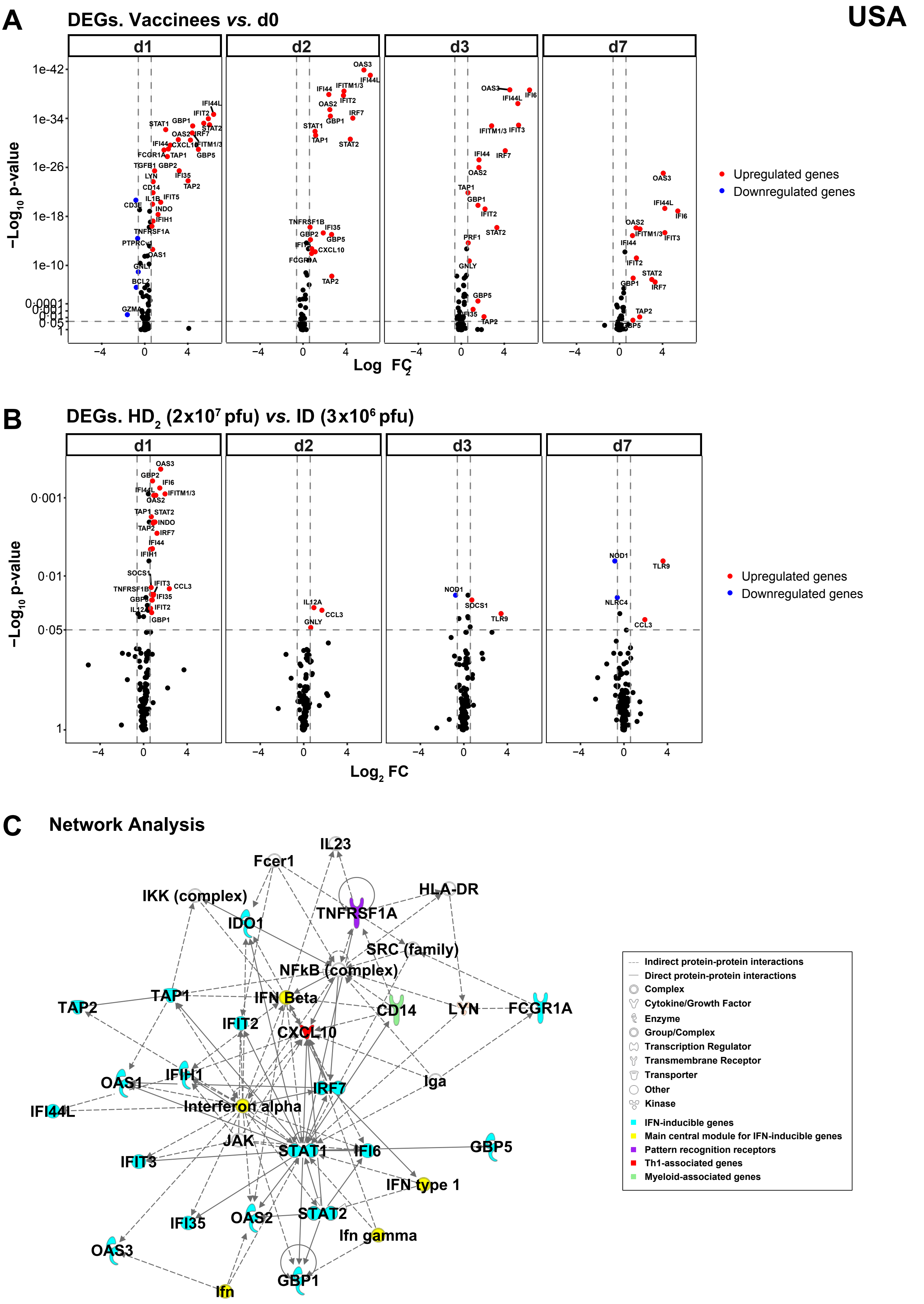
A
DEGs. Vaccinees vs. d0
USA
d1
d2
d3
d7
–Log10 p-value
Log2 FC
Upregulated genes
Downregulated genes
B
DEGs. HD2 (2x10^7 pfu) vs. ID (3x10^6 pfu)
d1
d2
d3
d7
–Log10 p-value
Log2 FC
Upregulated genes
Downregulated genes
C
Network Analysis
IL23
Fcer1
HLA-DR
IKK (complex)
TNFRSF1A
IDO1
SRC (family)
NFkB (complex)
TAP2
TAP1
IFN Beta
CD14
LYN
FCGR1A
IFIT2
CXCL10
OAS1
IFIH1
IRF7
Iga
IFI44L
Interferon alpha
JAK
STAT1
IFI6
GBP5
IFIT3
IFN type 1
IFI35
OAS2
STAT2
Ifn gamma
OAS3
GBP1
Ifn
Indirect protein-protein interactions
Direct protein-protein interactions
Complex
Cytokine/Growth Factor
Enzyme
Group/Complex
Transcription Regulator
Transmembrane Receptor
Transporter
Other
Kinase
IFN-inducible genes
Main central module for IFN-inducible genes
Pattern recognition receptors
Th1-associated genes
Myeloid-associated genes

Figure S7. Identification of differentially expressed genes (DEGs) and key networks following rVSVΔG-ZEBOV-GP vaccination in the USA cohort. Differential Expression Analysis was performed on GAPDH-normalized log_2-transformed gene expression data of the USA cohort. (A) Volcano plots representing DEGs at different timepoints (d1, d2, d3, and d7) following rVSVΔG-ZEBOV-GP vaccination of all vaccinees compared to their baseline gene expression values. The y-axis scales of all plots are harmonized. P-values, $-log_{10}$-transformed for a better visualization, are plotted against log_2 FC. Genes with p <0·05 and log_2 FC <-0·6 or >0·6 were labelled as DEGs. (B) Volcano plots representing DEGs at different timepoints (d1, d2, d3, and d7) following rVSVΔG-ZEBOV-GP vaccination comparing vaccinees that received a high-dose ($2x10^7$ pfu) versus the ID ($3x10^6$ pfu). The HD_2 dose was selected for the comparison since this dose is currently used for routine clinical application and this dose is shared with the other 3 cohorts. The y-axis scales of all plots are harmonized. P-values, $-log_{10}$-transformed for a better visualization, are plotted against log_2 FC. Genes with p <0·05 and log_2 FC <-0·6 or >0·6 were labelled as DEGs. (C) Network analysis on the DEGs identified between d0 and d1 following rVSVΔG-ZEBOV-GP vaccination of all vaccinees compared to their baseline gene expression levels was performed using Ingenuity Pathway Analysis (IPA).

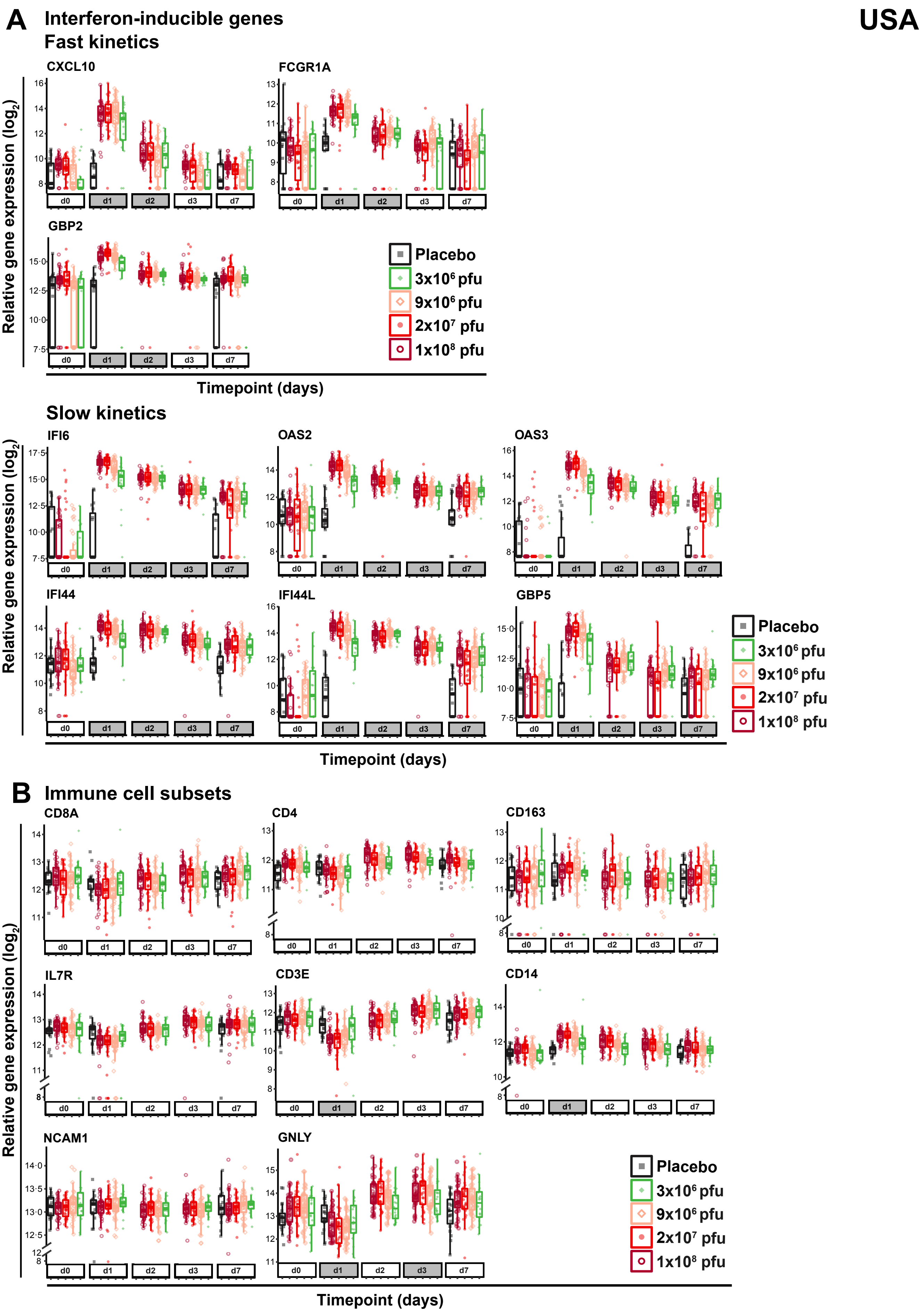

A Interferon-inducible genes
Fast kinetics
USA
CXCL10
FCGR1A
GBP2
Relative gene expression (log2)
Placebo
3x10⁶ pfu
9x10⁶ pfu
2x10⁷ pfu
1x10⁸ pfu
d0
d1
d2
d3
d7
Timepoint (days)
Slow kinetics
IFI6
OAS2
OAS3
IFI44
IFI44L
GBP5
B Immune cell subsets
CD8A
CD4
CD163
IL7R
CD3E
CD14
NCAM1
GNLY

Figure S8. Expression kinetics of representative genes in the USA cohort following rVSVΔG-ZEBOV-GP vaccination. Log_2-transformed relative gene expression values of (A) interferon-inducible genes displaying either fast (top panel) or slow (bottom panel) kinetics and (B) immune cell subset genes. Box plots depict median gene expression values and the inter quartile range (IQR), while the whiskers represent the data within the Q_1-1·5xIQR and Q_3+1·5xIQR interval. Outliers are reported as symbols. Vaccinees receiving either a HD_3 ($1x10^8$ pfu), HD_2 ($2x10^7$ pfu), HD_1 ($9x10^6$ pfu), ID ($3x10^6$ pfu), or placebo control (P) are shown in distinct colors. Timepoints post-vaccination at which genes are differentially expressed compared to baseline are indicated in grey. Statistical significance ($p < 0·05$) was determined using a non-parametric Mann-Whitney test with Benjamini-Hochberg correction for multiple testing.

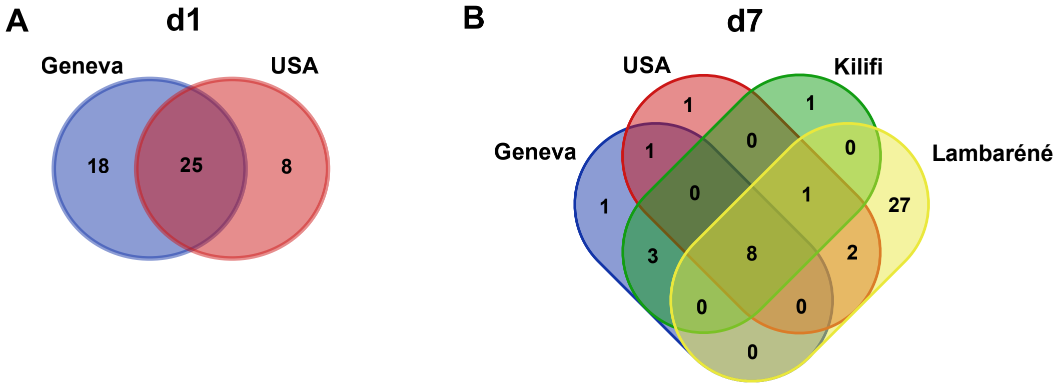

Figure S9. Venn diagram depicting DEGs shared between the different study cohorts. The number of DEGs shared between the Geneva and USA cohort at d1 (A) and all 4 study cohorts at d7 (B) are depicted in a Venn diagram.

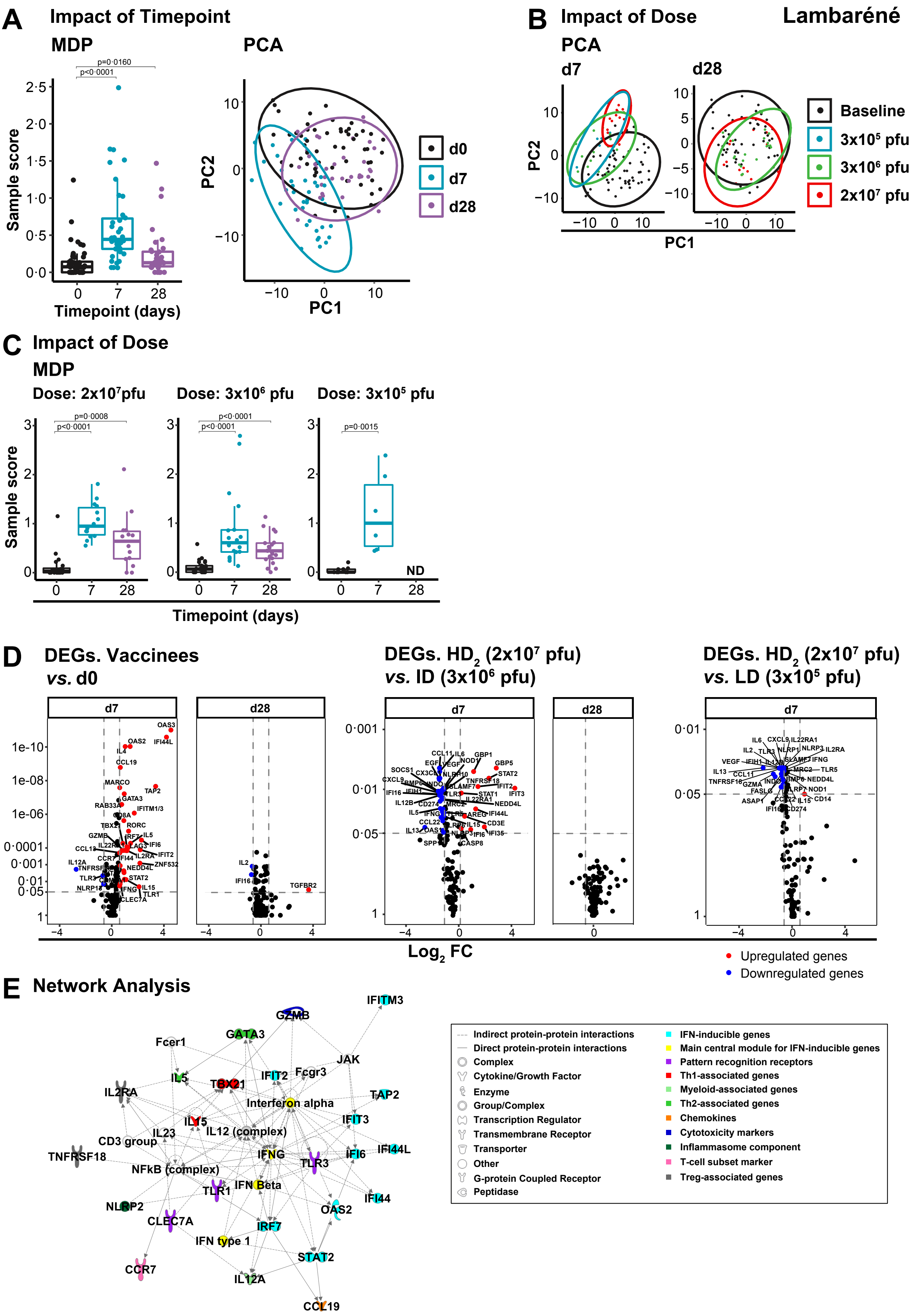
A Impact of Timepoint
MDP
PCA
p=0·0160
p<0·0001
Sample score
Timepoint (days)
PC1
PC2
d0
d7
d28
B Impact of Dose
Lambaréné
PCA
d7
d28
PC1
PC2
Baseline
3x10⁵ pfu
3x10⁶ pfu
2x10⁷ pfu
C Impact of Dose
MDP
Dose: 2x10⁷pfu
Dose: 3x10⁶ pfu
Dose: 3x10⁵ pfu
p=0·0008
p<0·0001
p=0·0015
ND
Sample score
Timepoint (days)
D DEGs. Vaccinees vs. d0
DEGs. HD₂ (2x10⁷ pfu) vs. ID (3x10⁶ pfu)
DEGs. HD₂ (2x10⁷ pfu) vs. LD (3x10⁵ pfu)
d7
d28
−Log₁₀ p-value
Log₂ FC
Upregulated genes
Downregulated genes
E Network Analysis
Indirect protein-protein interactions
Direct protein-protein interactions
Complex
Cytokine/Growth Factor
Enzyme
Group/Complex
Transcription Regulator
Transmembrane Receptor
Transporter
Other
G-protein Coupled Receptor
Peptidase
IFN-inducible genes
Main central module for IFN-inducible genes
Pattern recognition receptors
Th1-associated genes
Myeloid-associated genes
Th2-associated genes
Chemokines
Cytotoxicity markers
Inflammasome component
T-cell subset marker
Treg-associated genes

Figure S10. Impact of time and dose on gene expression profiles and identification of DEGs and key networks following rVSVΔG-ZEBOV-GP vaccination in the Lambaréné cohort. Molecular Degree of Perturbation (MDP), Principal Component Analysis (PCA), Differential Expression Analysis and IPA network analysis were performed on *GAPDH*-normalized log_2-transformed gene expression data of the Lambaréné cohort. (A) Samples were separated by timepoint (d0, d7, and d28) to evaluate the impact of time. d0 samples of vaccinees were used as baseline controls. Timepoints were compared using Mann-Whitney U test. (B) PCA analysis at distinct timepoints. Samples were separated per dose to evaluate the impact of the dose. d0 samples of vaccinees were used as baseline controls. (C) MDP analysis at distinct timepoints. Samples were separated per dose to evaluate the impact of the dose. d0 samples of vaccinees were used as baseline controls. Timepoints were compared using Mann-Whitney U test. (D) Volcano plots representing DEGs at different timepoints (d7 and 28) following rVSVΔG-ZEBOV-GP vaccination of all the vaccinees compared to their baseline gene expression values or comparing vaccinees that received a HD_2 ($2x10^7$ pfu) versus an ID ($3x10^6$ pfu) or LD ($3x10^5$ pfu). The y-axis scales of all plots are harmonized per comparison. P-values, $-log_{10}$-transformed for a better visualization, are plotted against log_2 FC. Genes with p <0·05 and log_2 FC <-0·6 or >0·6 were labelled as DEGs. (E) Network analysis on the DEGs identified between d0 and d7 following rVSVΔG-ZEBOV-GP vaccination of all vaccinees compared to their baseline gene expression levels was performed using Ingenuity Pathway Analysis (IPA). ND = Not determined because samples were not available.

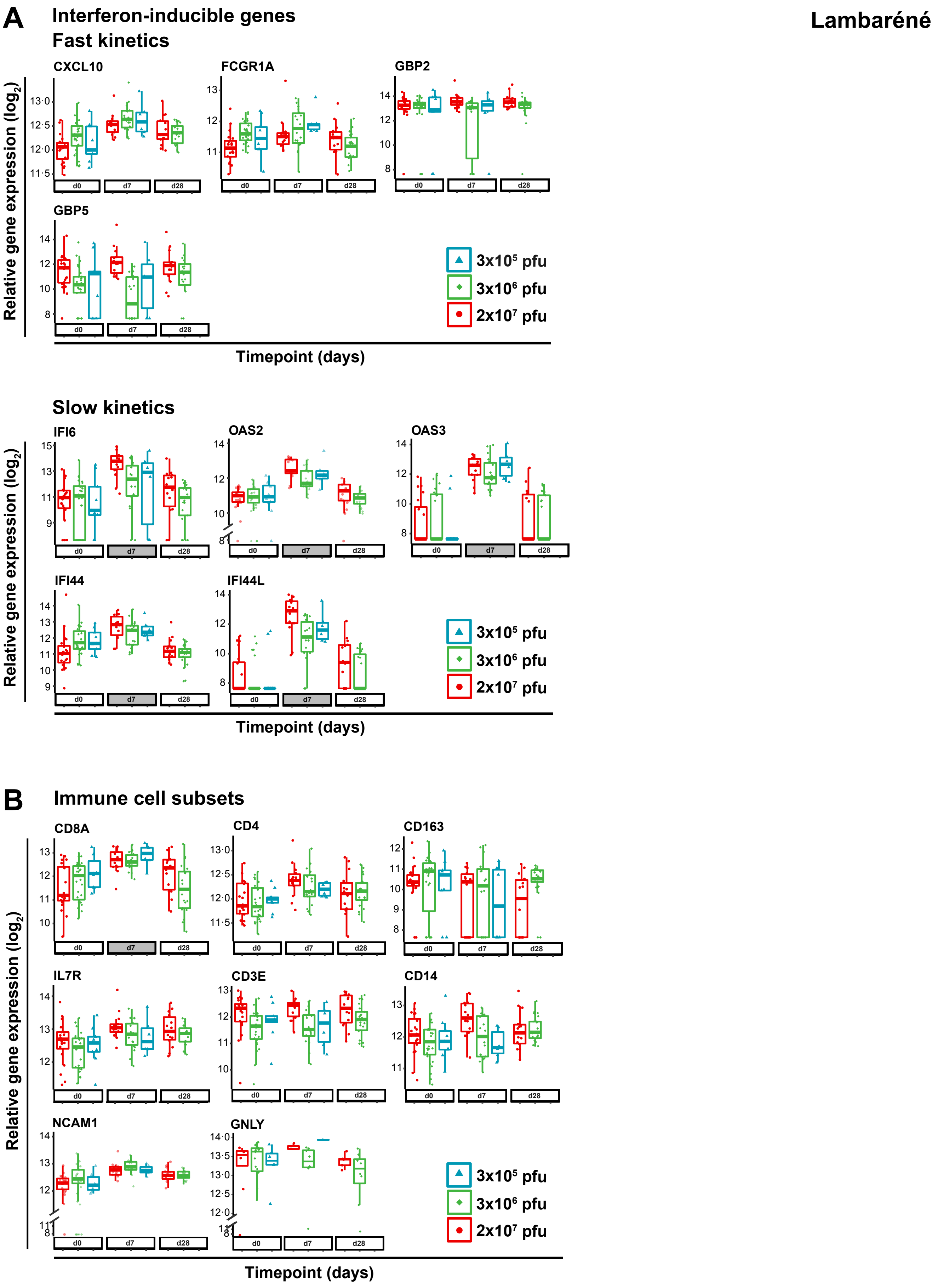
A
Interferon-inducible genes
Fast kinetics
Lambaréné
CXCL10
FCGR1A
GBP2
GBP5
Relative gene expression (log2)
d0
d7
d28
3x10⁵ pfu
3x10⁶ pfu
2x10⁷ pfu
Timepoint (days)
Slow kinetics
IFI6
OAS2
OAS3
IFI44
IFI44L
B
Immune cell subsets
CD8A
CD4
CD163
IL7R
CD3E
CD14
NCAM1
GNLY

Figure S11. Expression kinetics of representative genes in the Lambaréné cohort following rVSVΔG-ZEBOV-GP vaccination. Log_2-transformed relative gene expression levels of (A) interferon-inducible genes and (B) immune cell subset genes. Box plots depict median gene expression values and the inter quartile range (IQR), while the whiskers represent the data within the Q_1-1·5xIQR and Q_3+1 5xIQR interval. Outliers are reported as symbols. Vaccinees receiving either a HD_2 ($2x10^7$ pfu), ID ($3x10^6$ pfu), or LD ($3x10^5$ pfu) are represented by different colors. Timepoints post-vaccination at which genes are differentially expressed compared to baseline are indicated in grey. Statistical significance ($p <0·05$) was determined using a non-parametric Mann-Whitney test with Benjamini-Hochberg correction for multiple testing.

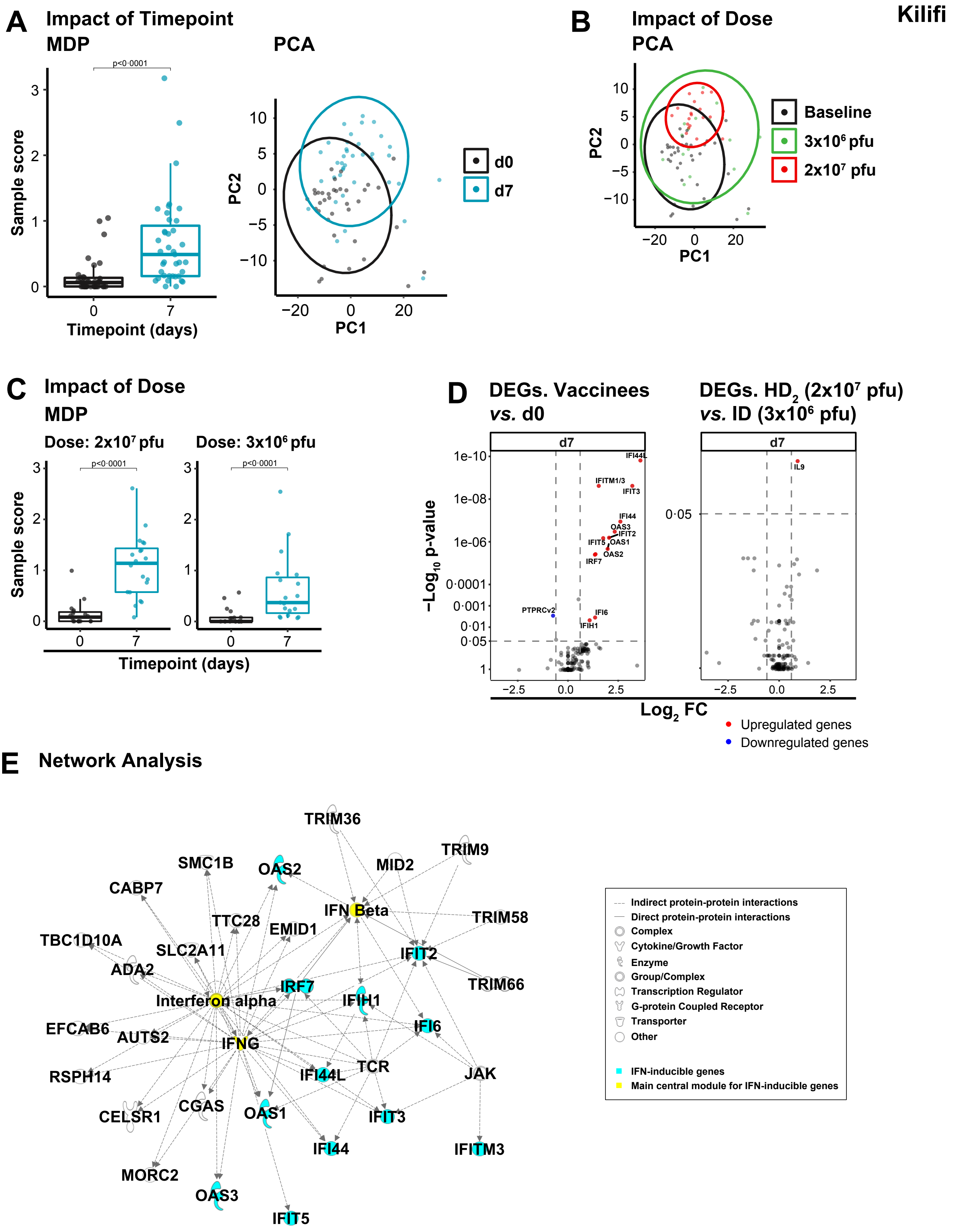
Kilifi
A
Impact of Timepoint
MDP
PCA
p<0·0001
Sample score
Timepoint (days)
PC2
PC1
d0
d7
B
Impact of Dose
PCA
Baseline
3x10⁶ pfu
2x10⁷ pfu
C
Impact of Dose
MDP
Dose: 2x10⁷ pfu
Dose: 3x10⁶ pfu
D
DEGs. Vaccinees vs. d0
DEGs. HD2 (2x10⁷ pfu) vs. ID (3x10⁶ pfu)
d7
−Log10 p-value
Log2 FC
IFI44L
IFITM1/3
IFIT3
IFI44
OAS3
IFIT2
IFIT5
OAS1
OAS2
IRF7
PTPRCv2
IFI6
IFIH1
IL9
Upregulated genes
Downregulated genes
E
Network Analysis
TRIM36
SMC1B
OAS2
MID2
TRIM9
CABP7
IFN Beta
TTC28
EMID1
TRIM58
TBC1D10A
SLC2A11
IFIT2
ADA2
IRF7
TRIM66
Interferon alpha
IFIH1
IFI6
EFCAB6
AUTS2
IFNG
RSPH14
IFI44L
TCR
JAK
CELSR1
CGAS
OAS1
IFIT3
IFI44
IFITM3
MORC2
OAS3
IFIT5
Indirect protein-protein interactions
Direct protein-protein interactions
Complex
Cytokine/Growth Factor
Enzyme
Group/Complex
Transcription Regulator
G-protein Coupled Receptor
Transporter
Other
IFN-inducible genes
Main central module for IFN-inducible genes

Figure S12. Impact of time and dose on gene expression profiles and identification of DEGs and key networks following rVSVΔG-ZEBOV-GP vaccination in the Kilifi cohort. Molecular Degree of Perturbation (MDP), Principal Component Analysis (PCA), Differential Expression Analysis, and IPA network analysis were performed on *GAPDH*-normalized log_2-transformed gene expression data of the Kilifi cohort. (A) Samples were separated by timepoint (d0, and d7) to evaluate the impact of time. d0 samples of vaccinees were used as baseline controls. Timepoints were compared using Mann-Whitney U test. (B) PCA analysis at d7. Samples were separated per dose to evaluate the impact of the dose. d0 samples of vaccinees were used as baseline controls. (C) MDP analysis at distinct timepoints. Samples were separated per dose to evaluate the impact of the dose. d0 samples of vaccinees were used as baseline controls. Timepoints were compared using Mann-Whitney U test. (D) Volcano plots representing differentially expressed genes at d7 following rVSVΔG-ZEBOV-GP vaccination of all the vaccinees versus their baseline gene expression values or comparing vaccinees that received a HD_2 ($2x10^7$ pfu) versus an ID ($3x10^6$ pfu). P-values, $-log_{10}$-transformed for a better visualization, are plotted against log_2 FC. Genes with p <0·05 and log_2 FC <-0·6 or >0·6 were labelled as DEGs. (E) Network analysis on the DEGs identified between d0 and d7 following rVSVΔG-ZEBOV-GP vaccination of all vaccinees compared to their baseline gene expression levels was performed using Ingenuity Pathway Analysis (IPA).

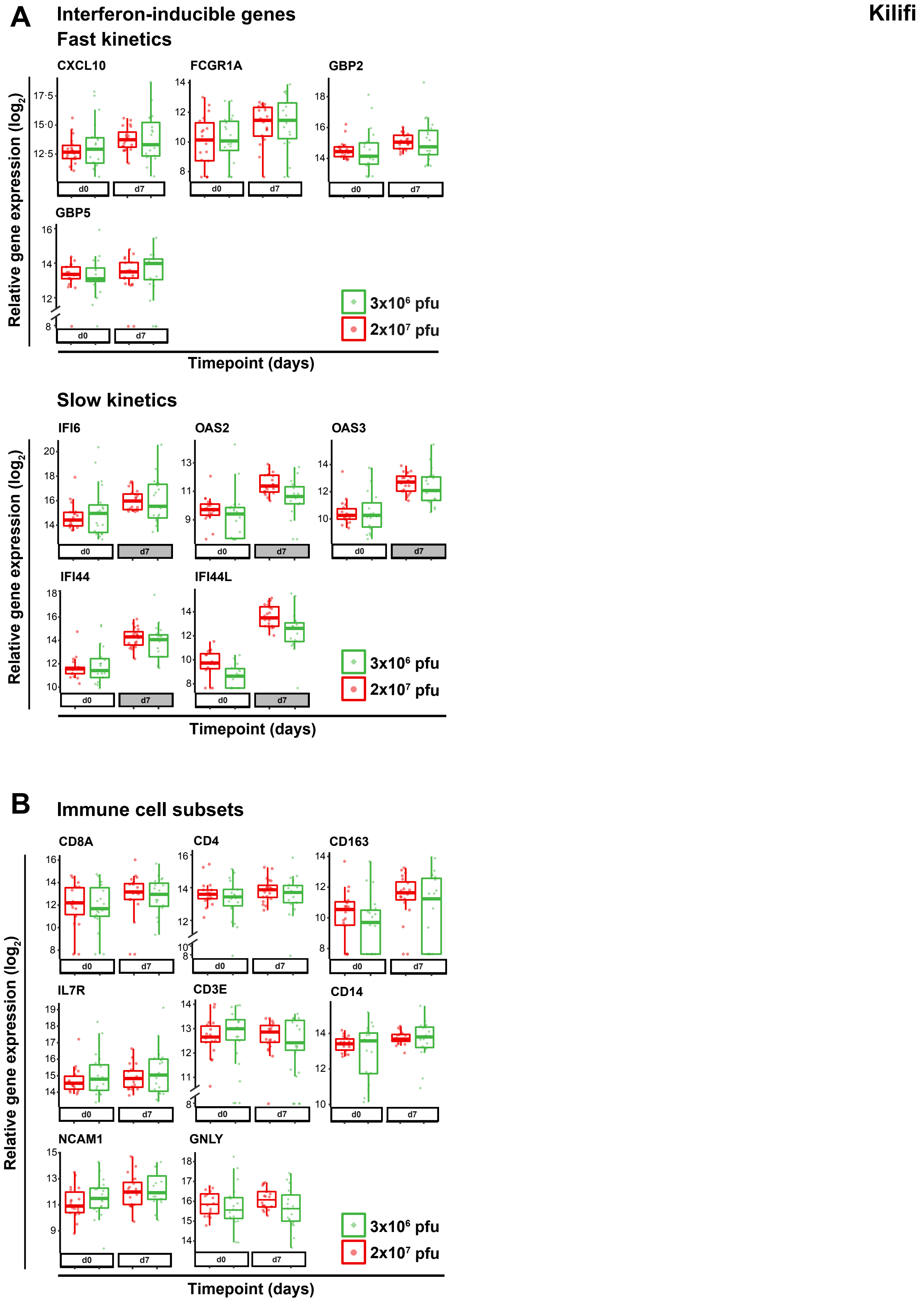
A
Interferon-inducible genes
Fast kinetics
Kilifi
CXCL10
FCGR1A
GBP2
GBP5
Relative gene expression (log_2)
d0
d7
3x10^6 pfu
2x10^7 pfu
Timepoint (days)
Slow kinetics
IFI6
OAS2
OAS3
IFI44
IFI44L
B
Immune cell subsets
CD8A
CD4
CD163
IL7R
CD3E
CD14
NCAM1
GNLY

Figure S13. Expression kinetics of representative genes in the Kilifi cohort following rVSVΔG-ZEBOV-GP vaccination. Log_2-transformed relative transcript levels of (A) interferon-inducible genes and (B) immune cell subset genes. Box plots depict median gene expression values and the inter quartile range (IQR), while the whiskers represent the data within the Q_1-1·5xIQR and Q_3+1·5xIQR interval. Outliers are reported as symbols. Vaccinees receiving either a HD_2 ($2x10^7$ pfu) or ID ($3x10^6$ pfu) are represented in distinctive colors. Timepoints post-vaccination at which genes are differentially expressed compared to baseline are indicated in grey. Statistical significance ($p < 0·05$) was determined using a non-parametric Mann-Whitney test with Benjamini-Hochberg correction for multiple testing.

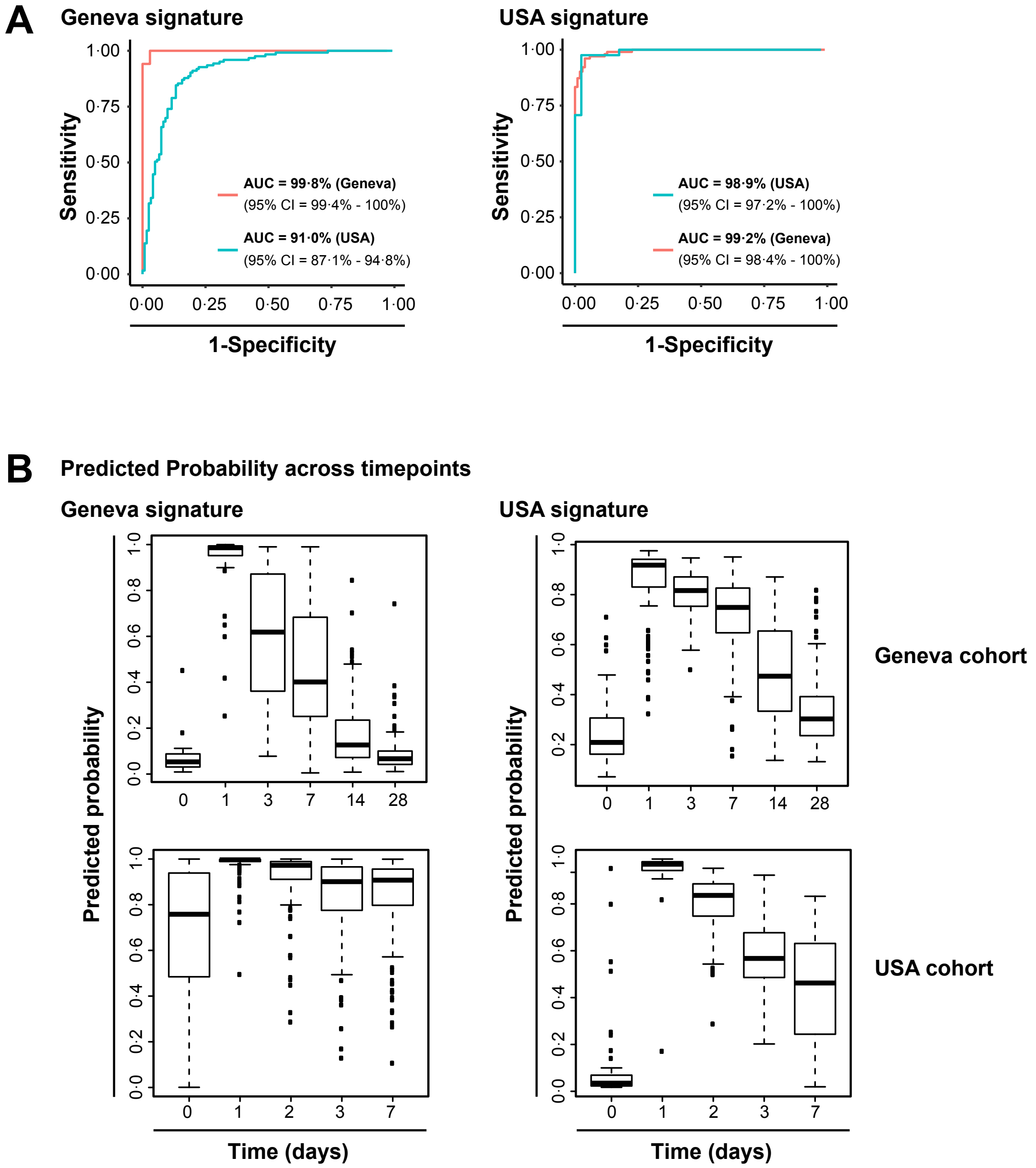

Figure S14. Identification of signatures associated with rVSVΔG-ZEBOV-GP vaccination at the peak of the transcriptomic response (d1) in the Geneva and USA cohorts. Geneva (left panels) or USA (right panels) transcriptomic datasets were used to train the models. 70% of each dataset was used as training set, while 30% was used as test set. (A) Receiver Operating Characteristic (ROC) curves (Sensitivity plotted against 1-Specificity) and Area Under the Curve (AUC) showing the classifying performance of the trained models. The model trained on 70% of the Geneva dataset was tested in the remaining 30% of the Geneva dataset (left panel, red line) and validated using the complete dataset of USA cohort (left panel, blue line). Vice versa, the model trained on 70% of the USA dataset was tested in the remaining 30% of the USA dataset (right panel, blue line) and validated using the complete dataset of Geneva cohort (right panel, red line). (B) Predicted probability plots showing the accuracy of identified biomarker signatures across timepoints in box-and-whiskers plots (5-95 percentiles) either in the cohorts in which the Train Test Split (TTS) was performed or in the validation cohort.

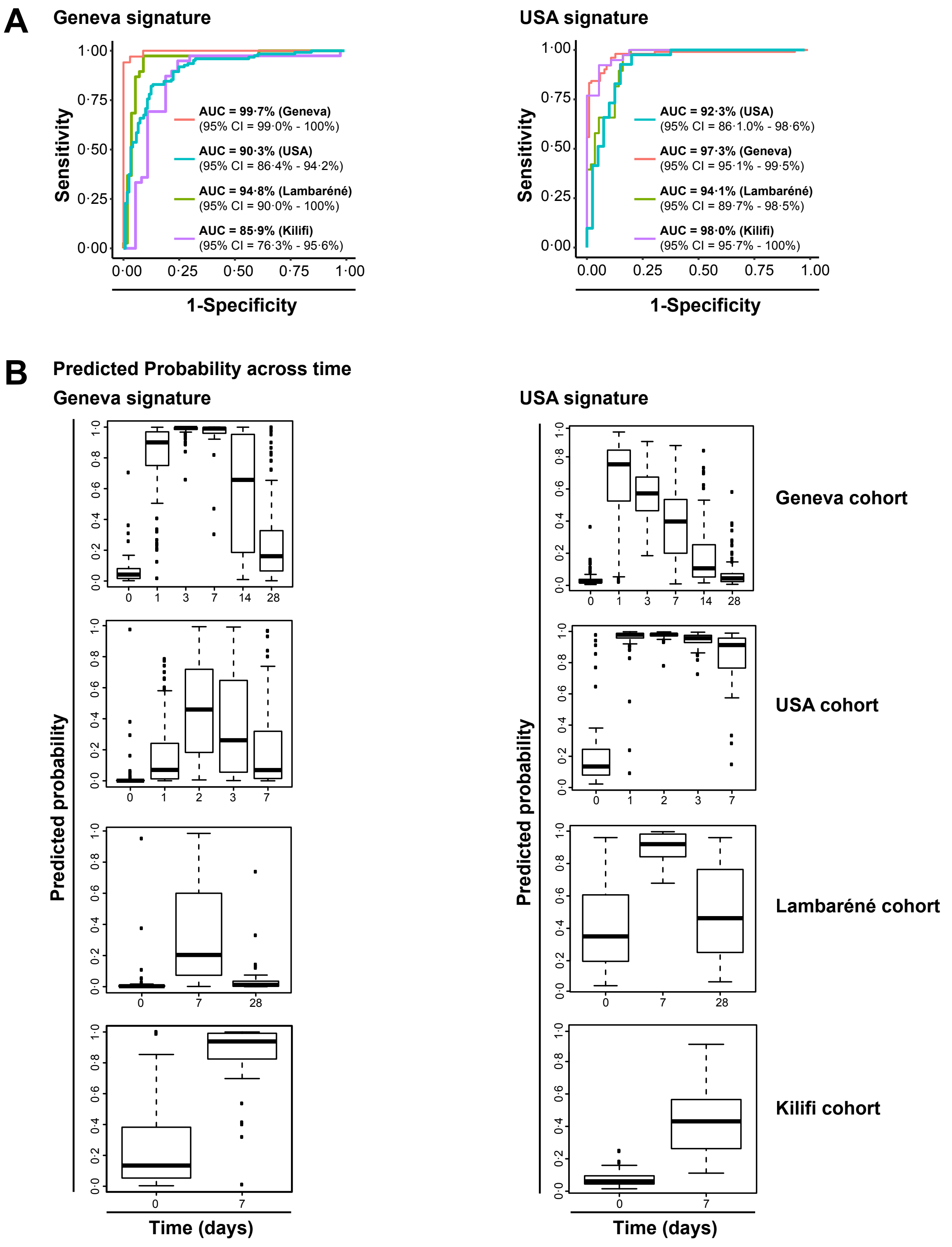

Figure S15. Identification of common signatures associated with rVSVΔG-ZEBOV-GP vaccination at d7 post-vaccination in all 4 study cohorts. Geneva (left panels) and USA (right panels) transcriptomic datasets were used to train the models: 70% of each dataset was used as train set, while 30% was used as test set. (A) Receiver Operating Characteristic (ROC) curves (Sensitivity plotted against 1-Specificity) and Area Under the Curve (AUC) showing the classifying performance of the trained models. The model trained on 70% of the Geneva dataset was tested in the remaining 30% of the Geneva dataset (left panel, red line) and validated using the complete datasets of USA (blue line), Lambaréné (green line), or Kilifi (purple line). The model trained on 70% of the USA dataset was tested in the remaining 30% of the USA dataset (right panel, blue line) and validated using the complete datasets of Geneva (red line), Lambaréné (green line), or Kilifi (purple line). (B) Predicted probability plots showing the accuracy of identified biomarker signatures across timepoints in box-and-whiskers plots (5-95 percentiles) either in the cohorts in which the Train Test Split (TTS) was performed or in the validation cohorts.

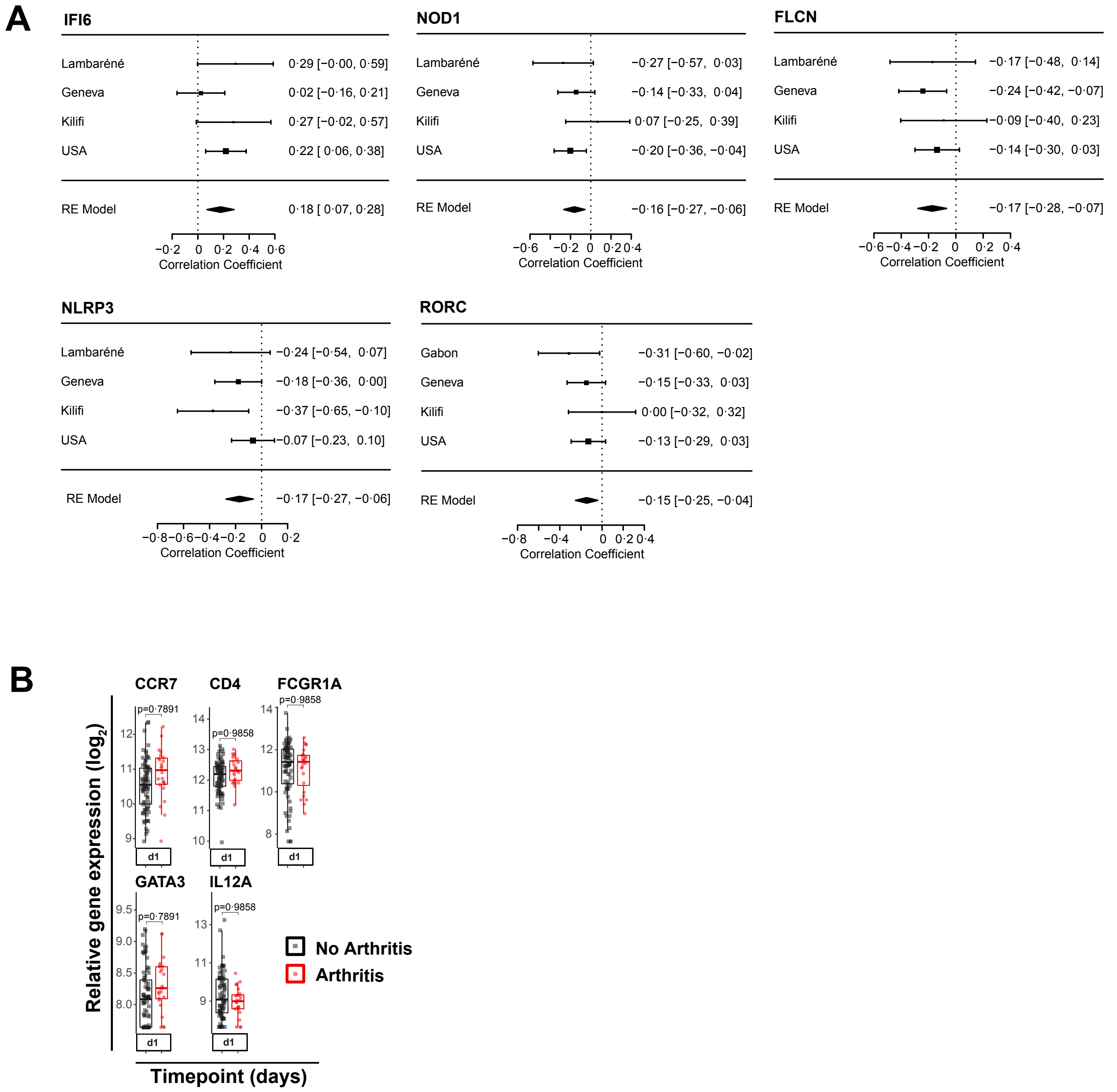

Figure S16. Correlation between gene expression profiles and ZEBOV-GP-specific antibody titers in response to rVSVΔG-ZEBOV-GP vaccination in cohorts from Europe, USA, and Africa and reactogenicity (arthritis) in the Geneva cohort

(A) Forest plots of the five-gene signature (*IFI6*, *NOD1*, *FLCN*, *NLRP3*, and *RORC*). Log_2 FC and their corresponding 95% confidence interval (x-axis) are plotted for each cohort (y-axis). The rhombus represents log_2 FC summaries while on the right side of each plot the log_2 FC summaries and their 95% confidence intervals are shown. (B) Log_2-transformed relative gene expression levels of the single genes encompassing the 5-gene signature at d1 post-vaccination. Box plots depict median gene expression values and the inter quartile range (IQR), while the whiskers represent the data within the Q_1-1·5xIQR and Q_3+1 5xIQR interval. Outliers are reported as symbols. Mann-Whitney U test with Benjamini-Hochberg correction for multiple testing have been applied to assess the significant difference between arthritis and no arthritis.

Appendix 10. Supplementary References

1. Geluk A, van Meijgaarden KE, Wilson L, et al. Longitudinal immune responses and gene expression profiles in type 1 leprosy reactions. *J Clin Immunol* 2014; **34**(2): 245-55.
2. Joosten SA, Goeman JJ, Sutherland JS, et al. Identification of biomarkers for tuberculosis disease using a novel dual-color RT-MLPA assay. *Genes Immun* 2012; **13**(1): 71-82.
3. Team RC. A language and environment for statistical computing. R Foundation for Statistical Computing: R Foundation for Statistical Computing; 2018.
4. Lever M, Russo P, Nakaya H. mdp: Molecular Degree of Perturbation calculates scores for transcriptome data samples based on their perturbation from controls. *Bioconductor: Open sourse software for bioinformatics* 2019.
5. Pankla R, Buddhisa S, Berry M, et al. Genomic transcriptional profiling identifies a candidate blood biomarker signature for the diagnosis of septicemic melioidosis. *Genome Biol* 2009; **10**(11): R127.
6. Friedman J, Hastie T, Tibshirani R. Regularization Paths for Generalized Linear Models via Coordinate Descent. *J Stat Softw* 2010; **33**(1): 1-22.
7. Goeman JJ. Penalized R package, version 0.9-51. 2018.
8. Goeman JJ. L1 penalized estimation in the Cox proportional hazards model. *Biometrical Journal* 2010; **52**(1): 70-84.
9. Huttner A, Dayer JA, Yerly S, et al. The effect of dose on the safety and immunogenicity of the VSV Ebola candidate vaccine: a randomised double-blind, placebo-controlled phase 1/2 trial. *Lancet Infect Dis* 2015; **15**(10): 1156-66.
10. Heppner DG, Jr., Kemp TL, Martin BK, et al. Safety and immunogenicity of the rVSVG-ZEBOV-GP Ebola virus vaccine candidate in healthy adults: a phase 1b randomised, multicentre, double-blind, placebo-controlled, dose-response study. *Lancet Infect Dis* 2017; **17**(8): 854-66.
11. Agnandji ST, Huttner A, Zinser ME, et al. Phase 1 Trials of rVSV Ebola Vaccine in Africa and Europe. *N Engl J Med* 2016; **374**(17): 1647-60.
12. Viechtbauer W. Conducting Meta-Analyses in R with the metafor Package. *Journal of Statistical Software* 2010; **36**(3): 1-48.
13. Darst BF. Using recursive feature elimination in random forest to account for correlated variables in high dimensional data. *BMC Genetics* 2018; **19**: 65.
14. Kuhn M. Building Predictive Models in R Using the caret Package. *Journal of Statistical Software* 2008; **28**(5): 1-26.
